# Iron Deficiency Leads to Chlorosis Through Impacting Chlorophyll Synthesis and Nitrogen Metabolism in *Areca catechu* L.

**DOI:** 10.3389/fpls.2021.710093

**Published:** 2021-08-02

**Authors:** Jia Li, Xianmei Cao, Xiaocheng Jia, Liyun Liu, Haowei Cao, Weiquan Qin, Meng Li

**Affiliations:** ^1^Coconut Research Institute, Chinese Academy of Tropical Agricultural Sciences, Wenchang, China; ^2^Hainan Key Laboratory for Sustainable Utilization of Tropical Bioresources, College of Tropical Crops, Hainan University, Haikou, China; ^3^College of Life Science and Technology, Central South University of Forestry and Technology, Changsha, China

**Keywords:** iron, chlorosis, chloroplast, nitrogen metabolism, *Areca catechu* L.

## Abstract

Deficiency of certain elements can cause leaf chlorosis in *Areca catechu* L. trees, which causes considerable production loss. The linkage between nutrient deficiency and chlorosis phenomenon and physiological defect in *A. catechu* remains unclear. Here, we found that low iron supply is a determinant for chlorosis of *A. catechu* seedling, and excessive iron supply resulted in dark green leaves. We also observed morphological characters of *A. catechu* seedlings under different iron levels and compared their fresh weight, chlorophyll contents, chloroplast structures and photosynthetic activities. Results showed that iron deficiency directly caused chloroplast degeneration and reduced chlorophyll synthesis in chlorosis leaves, while excessive iron treatment can increase chlorophyll contents, chloroplasts sizes, and inflated starch granules. However, both excessive and deficient of iron decreases fresh weight and photosynthetic rate in *A. catechu* seedlings. Therefore, we applied transcriptomic and metabolomic approaches to understand the effect of different iron supply to *A. catechu* seedlings. The genes involved in nitrogen assimilation pathway, such as *NR* (nitrate reductase) and *GOGAT* (glutamate synthase), were significantly down-regulated under both iron deficiency and excessive iron. Moreover, the accumulation of organic acids and flavonoids indicated a potential way for *A. catechu* to endure iron deficiency. On the other hand, the up-regulation of POD-related genes was assumed to be a defense strategy against the excessive iron toxicity. Our data demonstrated that *A. catechu* is an iron-sensitive species, therefore the precise control of iron level is believed to be the key point for *A. catechu* cultivation.

## Introduction

*A. catechu* is one of the most important tropical industrial crops. It is mainly distributed in Asian countries such as China, India, Malaysia and Indonesia. Extensive documents have demonstrated that betel nut, the fruit of *A. catechu*, has various pharmacological activity, including antiparasitic, antibacterial, antifungal, anti-inflammatory, and analgesic effects. Therefore, betel nut is considered as not only a chewable snack but also a medicinal material to fight against diverse diseases (Gilani et al., 2004).

In China, more than 95% *A. catechu* cultivation area is located at Hainan province. In the past decades, the planting area of *A. catechu* in Hainan province has increased from 6.66 × 10^4^ acres to 2.85 × 10^5^ acres (from 2010 to 2019) (Public data of government work report, 2020). The total output of betel nut in 2019 is 2.87 × 10^5^ tons, and the total value is $ 4.92 × 10^9^, which accounts for 7.1% of the Gross Domestic Product (GDP) of Hainan province (Li et al., 2020). Therefore, the development of *A. catechu* industry is vital to the local economy. However, in recent years, leaf chlorosis in *A. catechu* was frequently reported in almost all planting areas in Hainan province, and the proportion of yellowing trees keeps increasing. The latest survey indicated that on average, 4.7% of the current *A. catechu* showed chlorosis symptoms, which will seriously reduce the yield of betel nut (unpublished data). Our previous study demonstrated that the yellowing trees were not infected by pathogens or virus, thus the chlorosis was assumed to be induced by physiological factors. It has been extensively documented that mineral nutrient deficiency or heavy metal stress could lead to physiological chlorosis (van Maarschalkerweerd and Husted, 2015). Leaf chlorosis could be caused by the deficiency of nitrogen, potassium, magnesium or iron in diverse species, but the mechanism might be different (Guerinot and Yi, 1994; Sun et al., 2017; Ueno et al., 2018; de Bang et al., 2020). In *A. catechu*, low levels of nitrogen, phosphorus, potassium, magnesium or zinc, and high calcium/magnesium ratio were reported to cause leaf chlorosis (Mathai, 1986; Cpcri, 1990; Chowdappa et al., 2002). However, *A. catechu* plants showed relatively strong resistance to nutrient deficiency during the cultivation, and these cases were occasional events and did not cause serious consequences. The determinant of the large-scale physiological chlorosis of *A. catechu* remains unclear.

Iron deficiency-induced chlorosis is a common phenomenon in many crops. It was estimated that more than 30% of the crops growing worldwide were threatened by iron deficiency (Imsande, 1998). Iron deficiency is mainly caused by the insoluble ferric hydroxide, the main existing form of iron, in soil, especially calcareous soil (Vose, 1982). The insoluble form of iron cannot be used by plants; thus, the bioavailability is seriously limited (Guerinot and Yi, 1994). For example, the typical symptom caused by iron deficiency is yellowing leaves with green veins in citrus (Fu et al., 2017). Moreover, iron deficiency is often related to the nutrient deficiency of other elements. In Chickpea, leaf chlorosis caused by reduced iron absorption, which was regulated by phosphorus. The phosphates decrease the solubility of iron oxide thus resulted in iron deficiency (Sánchez-Rodríguez et al., 2014).

There are two strategies developed by plants to dissolve and transport iron to adapt the iron deficiency stress (Marschner and Römheld, 1994). One strategy is adopted by dicotyledons and non-gramineae monocotyledons, which reduce ferric ion in soil to ferrous ion by ferric-chelate reductase (FCR) proteins encoded by the *FRO* genes, and they take up iron with transporters encoded by *IRT* genes (Kobayashi et al., 2010). Another strategy was used by most Gramineae species, which secrete iron carrier (PS) to the soil through their roots, and directly integrate ferric ions to absorb it (Kobayashi et al., 2010; Nozoye et al., 2011). For the plant species adopted the first strategy, phenols are often the main components of root exudates in response to iron deficiency (Romheld and Maschner, 1986; Susín et al., 1996; Curie and Briat, 2003; Hell and Stephan, 2003). The phenol compounds have multiple functions including iron chelation and reduction, free radical scavenging, antibacterial activity, and react as a carbon source for microbial growth (Rice-Evans and Miller, 1996; Cao et al., 1997; Blum et al., 2000). These evidences indicated that specific genes, enzymes and metabolites were involved into the response pathway of iron deficiency.

In this study, we identified that iron is a main determinant for physiological chlorosis of *A. catechu*. Thus, we performed comparative analysis at transcription and metabolism level to identify key genes and key metabolites involved in the iron deficiency-induced chlorosis pathway. Our data will provide a theoretical basis to understand the mechanism underlying physiological chlorosis of *A. catechu*, and to improve the adaptability and vitality of *A. catechu* seedlings.

## Materials and Methods

### Plant Materials and Iron Treatments

The seedlings of an *A. catechu* cultivar “Reyan NO.1,” bredby the Coconut Research Institute of Chinese Academy of TropicalAgricultural Sciences, were used in this study. All seedlings were grown at the *A. catechu* nursery of the Coconut Research Institute (19′33′N, 110°47′E). The *A. catechu* seedings with three fully expanded leaves (4-month-old) were selected, and each seedling was planted in a 16 cm × 22 cm plastic pot containing 1 kg medium consists of silica sand, perlite and vermiculite with the proportion 1:1:1 (v/v/v). The seedlings were grown in an artificial growth chamber with a constant photoperiod (16 h light/8 h darkness) and an average temperature of 27°C.

An special nutrient solution (Li J. et al., 2019) for *A. catechu* (10 mmol/L NH_4_NO_3_, 0.625 mmol/L KH_2_PO_4_, 9 mmol/L KNO_3_, 1.5 mmol/L CaCl_2_, 0.015 mmol/L ZnSO_4_, 0.75mmol/L MgSO_4_, 0.05 mmol/L H_3_BO_3_, 2.5 μmol/L KI, 0.05 mmol/L MnSO_4_.H_2_O, 0.05 μmol/L CuSO_4_, 0.5 μmol/L Na_2_MoO_4_, 0.5 μmol/L CoCl_2_), and 0.5 μM (iron deficiency, ID), 50 μM (normal, CK) and150 μM (excessive iron, EI) Fe-EDTA was applied in this study. The pH of all the nutrient solutions was adjusted to 5.8 with 0.1 mol/L KOH. Totally 20 *A. catechu* seedlings were prepared for each treatment. During the treatment, 400 ml nutrient solution was added to each pot every 3 days, and the excess salt was scavenged with deionized water every 10 days to prevent salt accumulation. The leaf samples of *A. catechu* seedlings were collected at 28 days after treatment, because the obvious chlorosis symptom could be observed at this phase in the seedlings grown under ID. Totally 10 youngest leaves were collected for each sample, which was then mixed and divided into two parts. One part was used for biochemical and physiological measurement, and the other part was immediately frozen in liquid nitrogen and stored at −80°C for transcriptome and metabolome analysis.

### Photosynthetic Gas Exchange and Pigments Measurement

Five plants from each treatment were randomly selected for the measurement of gas exchange and pigment contents. Photosynthetic rates were measured through a CIARS-2 portable photosynthesis system (PP systems, Herts, United Kingdom) at an ambient CO_2_ concentration under a controlled light intensity of 993–1003 μmol/m^2*^s. The samples were measured at between 9 and 11 a.m. on a clear day with the leaf temperature is 28 ± 0.4°C and the relative humidity is 45 ± 1%. Photosynthetic rates were measured in fully expanded leaves of at least five individuals of each treatment, and each sample was measured for three times.

Fresh leaf tissues were collected and used to determine pigment content through a spectrophotometer according to the method of Arnon (1949). Briefly, 0.5 g of leaf tissue were cut into small pieces, marinated in 10 ml of 95% ethanol and held for 48 h in darkness. The supernatants were collected by centrifugation and analyzed using a 752 N UV/Vis Spectrophotometer at 665, 649 and 470 nm, respectively.

### Transmission Electron Microscopy (TEM) Observation

Leaf samples were cut into small pieces (1 mm × 1.2 mm), fixed in 2.5% (v/v) glutaraldehyde in 0.1 mol/L phosphate buffer (pH 7.4) at 4°C for 4 h, then rinsed and incubated overnight in a solution of 1% (w/v) OsO_4_ at 4°C. The mixtures were subsequently dehydrated using an ethanol series and infiltrated in a gradient series of epoxy resin, and then embedded in the Epon 812 resin. Thin sections were made with a Leica UC7 ultramicrotome, stained in the solution containing 2% (w/v) uranyl acetate and 10 mM lead citrate (pH 12), and the sections were observed and recorded with a HT7700 (Hitachi, Japan) transmission electron microscope.

### Leaf Ionomic Concentration Determination

The aerial parts and subterranean parts of *A. catechu* seedlings were harvested and dried at 70°C until their dry weight was unchanged (for at least 48 h). The total nitrogen was determined by an automatic azotometer (KT8200, FOSS, Sweden), through alkaline hydrolysis diffusion. Leaf P, S, K, Ca, Mg, Fe, Mn, Zn, Cu, and B content was assayed by inductively coupled plasma-atomic emission spectrometry (ICP-AES, IRIS-Advan type, Thermo, United States) after digestion with 1 mol/L HCl.

### Determination of Enzyme Activity Involved in Nitrogen Assimilation

The enzyme activities of nitrate reductase (NR, EC 1.7.99.4), glutamine synthetase (GS, EC 6.3.1.2) and glutamate synthase (GOGAT, EC 1.4.7.1) were determined in the leaves of *A. catechu* seedlings. NR activity was determined based on a previously described method (Yu and Zhang, 2012). Plant tissues was homogenized in a triturator with prechilled extraction medium containing 25 mM phosphate buffer (pH 7.5), 5 mM cysteine and 5 mM EDTA–Na_2_. The enzyme extract was added to assay mixture (KNO_3_–phosphate buffer and NADH). The mixture was incubated for 30 min at 30°C. The reaction was stopped by adding 1% 4-aminobenzene sulfonic acid and 0.2% 1-naphthylamine and left standing for 30 min for color development at 30°C. The absorbance of the supernatant was measured at 540 nm immediately; GS activity was assayed according to a previously described method (Yu and Zhang, 2012). Plant tissues were ground in 0.05 M Tris–HCl solution (pH 8.0) containing 2 mM MgSO_4_, 2 mM DTT and 0.4 M sucrose in an ice-cold mortar with a pestle. The crude enzyme extract was added to assay mixture (0.25 M imidazole–HCl buffer, 0.30 M sodium hydrogen glutamate, 30 mM ATP-Na, and 0.5 M MgSO_4_). The mixture was incubated for 5 min at 25°C. And then, hydroxylamine hydrochloride (a mixture of 1 M hydroxylamine hydrochloride and 1 M HCl, 1:1) was added and left standing for 15 min. The reaction was stopped by adding FeCl_3_ solution containing 10% FeCl_3_, 24% trichloroacetic acid and 50% HCl (1:1:1). The resulting supernatants were measured at 540 nm; and GOGAT activity was measured by the method described by Singh and Srivastava (1986). Tissues were ground and extracted with a medium containing 0.2 M sodium phosphate (pH 7.5), 2 mM EDTA, 50 mM KCI, 0.1% (v/v) β-mercaptoethanol and 0.5% (w/v) Triton X-100. The enzyme was assayed spectrophotometrically following the oxidation of NADH and using 2 control mixtures (minus 2-oxoglutarate and minus glutamine in each case). The assay mixture contained 0.4 ml 20 mM L-glutamine, 0.4 ml 5 mM 2-oxoglutarate, 1 mM EDTA (added in assay buffer), 0.1 ml 100 mM KCI, 0.6 ml 1 mM NADH, and 0.5 ml of the enzyme preparation in a final volume of 3.0 ml completed with 25 mM sodium phosphate (pH 7.5). The reaction was started by adding L-glutamine immediately following the enzyme preparation. The decrease in absorbance was recorded for 5 min at 340 nm in a double beam spectrophotometer. The amount of NADH oxidized was calculated from a standard curve of NADH.

### RNA-Seq Analysis

The leaf samples were collected from the *A. catechu* seedlings grown under ID, EI and CK conditions. Each treatment contains three individual samples as biological replicates. The total RNA was extracted using E.Z.N.A. Plant RNA Kit (Omega, R6827-01, United States). The quality of RNA, including degradation and contamination was monitored on 1% agarose gels. RNA concentration and integrity of the total RNA were measured using Nano Photometer^®^ Spectrophotometer (IMPLEN, CA, United States) and Agilent 2100 Bioanalyzer (Agilent Technologies, CA, United States), respectively.

Subsequently, the library preparations were sequenced on the Illumina HiSeq platform to generate raw data. After sequencing, the clean reads were obtained by removing reads containing adaptors, more than 5% unknown bases and low-quality reads (>20% of the bases with a quality score of ≤ 10). Gene function was annotated based on the following databases: NCBI non-redundant protein sequences (NR), clusters of orthologous (KOG/COG), gene ontology (GO), manually annotated and reviewed protein sequence database (Swiss-Prot), Kyoto Encyclopedia of Genes and Genomes (KEGG). Gene expression levels were represented using fragments per kilobase of transcript per million fragments mapped (FPKM) method. The differentially expressed genes (DEGs) were recruited based on False Discovery Rate (FDR) < 0.05 and | log2Fold Change| ≥ 1. All DEGs were analyzed by GO enrichment using GOseq (1.10.0) (Young et al., 2010) and KEGG enrichment using KOBAS software (Mao et al., 2005; Minoru et al., 2016).

### qPCR Analysis

The extracted RNA of normal and albinotic leaf samples were converted into cDNA using PrimeScript^TM^ RT reagent Kit for qPCR (RR047Q, TaKaRa, Japan). Then the cDNA was 10 × diluted and used as templates for qPCR. The qPCR reaction was performed using the PowerUp^TM^ SYBR^TM^ Green Master Mix (A25777, Applied Biosystems, United States) in an ABI real-time instrument (QuantStudio^TM^ 6 Flex System, United States). Three independent biological replicates of each sample with internal technical replicates were used for qPCR analysis. An *A. catechu* gene, *AcActin* (CL9155.Contig7) (Li et al., 2020) was used as the reference gene for data normalization. Primers used in qPCR are shown in Supplementary Table 1. The relative expression fold of each sample was calculated by its C_T_ value normalized to the C_T_ value of reference gene using the 2^–ΔΔCT^ method described by Livak and Schmittgen (2001). The normalized values of relative expression and FPKM values were calculated by log2, respectively, and the values were used to analyze the correlation between qPCR and RNA-seq results.

### Metabolite Profiling Analysis

The leaf samples were collected from the *A. catechu* seedlings grown under ID, EI and CK conditions for metabolome analysis. Each treatment contains three individual samples as biological replicates. Sample preparation, metabolite extraction and analysis were carried out as follows. In brief, the 100 mg freeze-dried samples were extracted overnight at 4°C with 1.0 ml 70% aqueous methanol (containing 0.1 mg/L lidocaine). Subsequently, 10,000 g centrifugation for 10 min at 4°C, then the extracts were absorbed and filtered before LC-MS analysis. A quality-control sample was prepared by equal blending of all samples; during the assay, the quality control sample was run every 10 injections to monitor the stability of the analytical conditions. The extracted samples were analyzed using a HPLC system (Shim-pack UFLC SHIMADZU CBM 30A system) equipped with Waters ACQUITY UPLC HSS T3 C18 column (1.8 μm, 2.1 mm × 100 mm). LIT and triple quadrupole (QQQ) scans were acquired on a triple quadrupole-linear ion trap mass spectrometer (Q TRAP), API 6500 Q TRAP LC/MS/MS System, equipped with an ESI Turbo Ion-Spray interface, operating in a positive ion mode and controlled by Analyst 1.6.3 software (AB Sciex). The solvent system, gradient program and ESI source operation parameters were carried out as described by previous research. The qualitative analysis of primary and secondary MS data was performed by searching the internal database using a self-compiled database MWDB (Metware Biotechnology Co., Ltd. Wuhan, China), Data pre-processing and metabolites identification were performed by the standard metabolic procedures, including comparing the m/z values, RT, and the fragmentation patterns with the standards. The variable importance of the projection (VIP) score of the application (O) PLS model was used to filter the best differentiated metabolites between treatments. Metabolites with significant differences in content were set with thresholds of variable importance in projection (VIP) ≥ 1 and fold change ≥ 2 or ≤ 0.5.

### Statistical Analysis

All data, including photosynthetic rates, pigment contents, enzyme activity and mineral nutrient concentration analysis were derived from three independent biological replicates. Data were analyzed using a one-way ANOVA analysis of variance, and differences between means were assessed using Duncan’s multiple range tests (*P* < 0.05). All statistical analyses were performed with SPSS version 19.0 (SPSS Inc., Chicago, United States).

## Results

### Morphological and Biochemical Characteristics of *A. catechu* Seedlings With Physiological Chlorosis

In order to clarify the main determinant of leaf chlorosis, we performed nutrient deficiency treatment of different elements, respectively. The results indicated that leaf chlorosis was not observed in the *A. catechu* seedlings grown under nitrogen and zinc deficiency until 80 days (Supplementary Figures 1, 2). Similarly, slight chlorosis appeared in the seedlings grown under the deficiency of boron, magnesium (Supplementary Figures 1, 3) and potassium (data not shown) after more than 80 days. The symptom of chlorosis could be recognized in 15 days and became obvious after 20-day ID treatment. The chlorosis generally initiated in the youngest leaf and aggravated in the following leaves, which exhibited gradually yellowing colors (Supplementary Figure 4). The yellowing seedlings grow very slowly, while keep alive but not develop for a long time. To clarify the mineral nutrient composition variation occurring in the yellowing leaves, we selected seven *A. catechu* planting areas for sampling and performing the element analysis. The yellowing or slight yellowing samples were found and collected in four of these planting areas. The results demonstrated that most mineral elements showed no difference between normal and yellowing leaves except for iron. The iron content was 58.14 and 58.37% on average in yellowing and slight yellowing leaves of that in normal ones, respectively (Supplementary Table 2).

**FIGURE 1:**
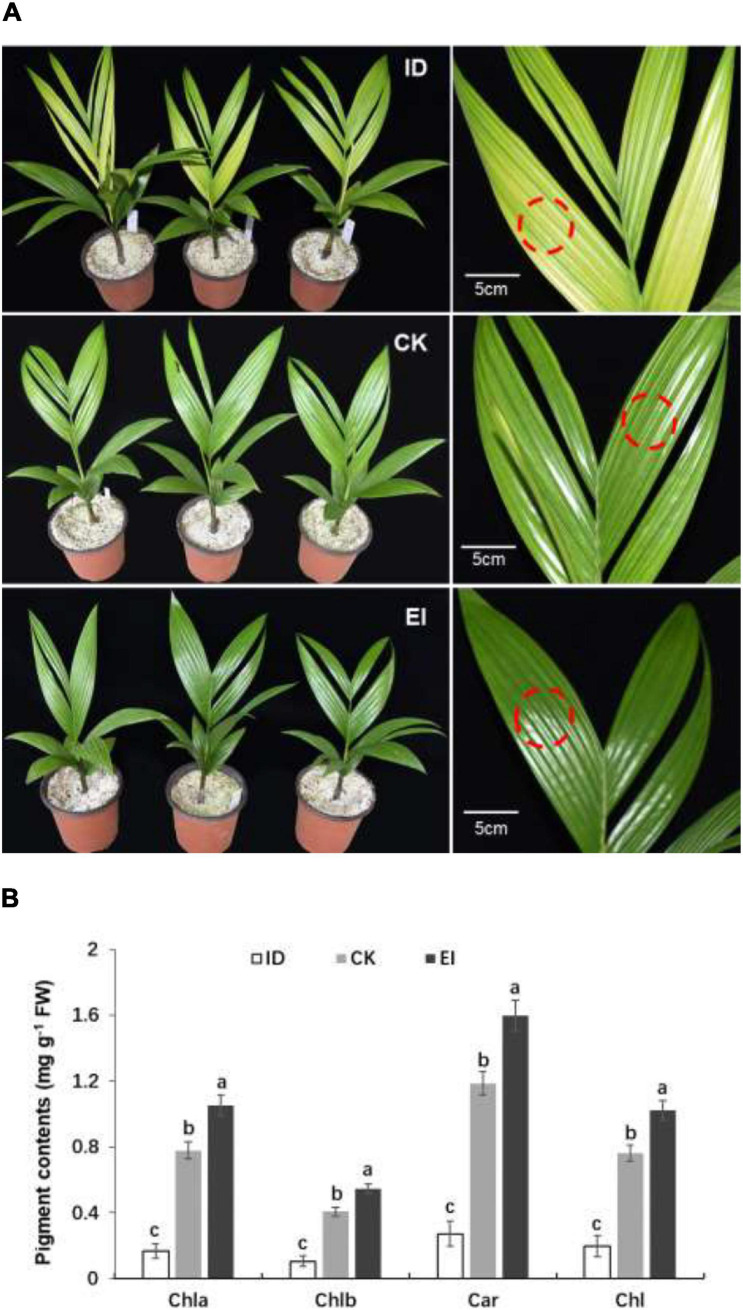
Morphological characteristics **(A)** and pigment content **(B)** of *A. catechu* seedlings grown under ID (0.5 μM Fe-EDTA), CK (50 μM Fe-EDTA), and EI (150 μM Fe-EDTA). ID, iron-deficiency treatment; CK, normal conditions; EI, excessive-iron treatment. Chla, chlorophyll *a*; Chlb, chlorophyll *b*; Car, carotenoid; Chl, chlorophyll *a* + chlorophyll *b*; red dashed circles represent the sampling areas. The small bars show standard deviation. Different letters represent significant differences at *P* < 0.05 according to Duncan’s multiple range tests.

Then the *A. catechu* seedlings were treated with different iron levels to investigate whether iron deficiency could directly induce physiological chlorosis. Obvious chlorosis symptom was observed in the seedlings grown under ID, and greener leaves were observed in the seedlings grown under EI (Figure 1A). The pigment contents were measured in normal and yellowing leaves. The results showed that all pigments, including chlorophyll *a*, chlorophyll *b* and carotenoids, were significantly reduced in the leaves under ID, and the pigment contents elevated along with the increasing iron levels, indicating that iron level is critical for pigment synthesis in *A. catechu* leaves (Figure 1B).

**FIGURE 2:**
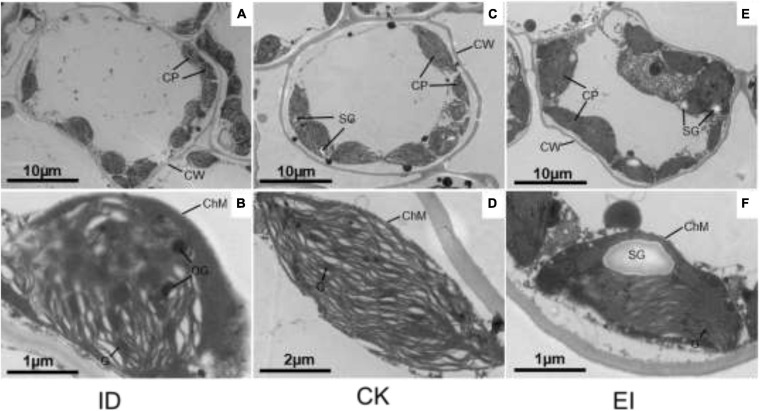
Chloroplast structures in ID, CK and EI leaves of *A. catechu*. **(A,B)** chloroplasts in ID leaf cells; **(C,D)** chloroplasts in CK leaf cells; **(E,F)** chloroplasts in EI leaf cells; CW, Cell wall; CP, Chloroplast; ChM, Chloroplast membrane; OG, Osmiophilic granule; SG, Starch grains.

The chloroplasts of the leaf cells under different treatments showed obvious structural variations. In the normal cells, the chloroplasts were well-developed, shuttle shaped with complete structure and close to the cell wall. The lamellae structure of grana and matrix was clear, and the thylakoids were stacked and arranged in order. On the contrary, partially disintegrated membrane was observed in the chloroplasts of the cells from yellowing leaves, the lamellae structure was imperfect or vanished, and a lot of osmiophagic granules were generated, indicating that ID seriously impacted the chloroplast structure. However, high iron level also impacted the lamellae structure, altered the chloroplasts into irregular shape, and produced more starch granules with larger size, implying that EI was also deleterious for *A. catechu* leaves (Figure 2).

**FIGURE 3:**
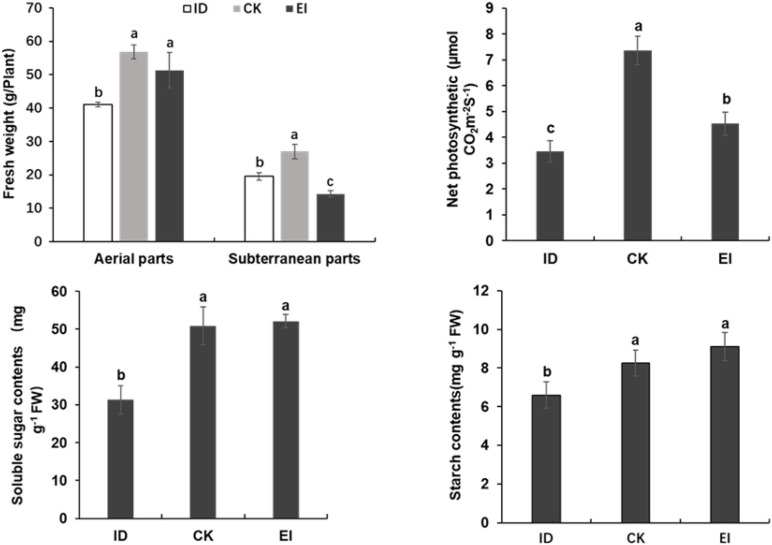
Fresh weight, photosynthetic efficiency and carbon hydrate in ID, CK and EI leaves. The small bars show standard deviation. Different letters represent significant differences at *P* < 0.05 according to Duncan’s multiple range tests.

The starch and soluble sugar content were significantly reduced in the leaves of *A. catechu* seedlings grown under ID, indicating that ID inhibited the photosynthesis and carbohydrate accumulation. However, seedlings grown under EI also showed reduced net photosynthetic rate, and reduced fresh weight in subterranean parts (Figure 3). This result might be attributed to the high iron toxicity, and indicated that the suitable concentration range of iron for *A. catechu* seedlings is narrow.

The mineral nutrient analysis demonstrated that the nitrogen, iron and manganese levels of *A. catechu* seedlings grown under ID were reduced in aerial part, and the nitrogen, potassium, magnesium and boron levels were reduced in subterranean part. While, the *A. catechu* seedlings grown under EI showed reduced nitrogen and iron levels in aerial part, and reduced potassium, magnesium and copper levels in subterranean part (Table 1). We noticed that iron level was accumulated and significantly elevated in the subterranean part of seedlings grown under EI. However, the iron level in the aerial part was reduced to the comparable level of ID, indicating that there is a mechanism of preventing excessive iron ion transport to the aerial part. This results also confirmed that high iron level is unacceptable for *A. catechu* seedlings.

**TABLE 1:** Nutrient concentration of aerial parts and subterranean parts in *A. catechu* under different treatments.

	**Aerial parts**			**Subterranean parts**		
	**ID**	**CK**	**EI**	**ID**	**CK**	**EI**
N (mg/g)	25.5 ± 0.6c	30.2 ± 1.2a	27.0 ± 1.5b	12.9 ± 1.1b	16.3 ± 0.1a	15.0 ± 0.5a
P (mg/g)	2.1 ± 0.3a	1.6 ± 0.2a	1.8 ± 0.1a	1.2 ± 0.0*a*b	1.1 ± 0.1b	1.3 ± 0.1a
S (mg/g)	3.5 ± 0.3a	3.2 ± 0.1a	3.2 ± 0.1a	4.0 ± 0.2a	3.4 ± 0.2b	4.1 ± 0.2a
K (mg/g)	27.6 ± 0.5a	24.1 ± 0.1b	25.1 ± 0.9b	19.2 ± 1.4b	26.6 ± 2.7a	21.6 ± 2.0b
Ca (mg/g)	8.2 ± 0.4a	5.2 ± 1.0b	5.1 ± 0.5b	9.0 ± 0.1a	8.1 ± 0.9b	11.8 ± 2.3a
Mg (mg/g)	1.9 ± 0.2a	1.7 ± 0.2*a*b	1.5 ± 0.1a	2.5 ± 0.1b	3.1 ± 0.2a	2.2 ± 0.1c
Fe (μg/g)	121.1 ± 3.2b	172.2 ± 16.3a	134.1 ± 7.7b	416.4 ± 23.2b	539.4 ± 105.2ab	655.0 ± 132.7a
Mn (μg/g)	53.9 ± 11.7b	61.5 ± 21.3ab	82.7 ± 16.2a	140.8 ± 16.7a	79.6 ± 6.8b	92.1 ± 7.4b
Zn (μg/g)	76.6 ± 6.7a	67.0 ± 6.1a	71.8 ± 15.6a	62.8 ± 4.2b	68.8 ± 1.1*a*b	71.6 ± 5.6a
Cu (μg/g)	6.5 ± 1.0a	7.4 ± 1.1a	6.3 ± 1.3a	25.5 ± 3.0a	19.9 ± 4.3b	12.5 ± 1.4c
B (μg/g)	1.9 ± 0.2a	2.2 ± 0.2a	2.2 ± 0.8a	0.3 ± 0.1b	2.2 ± 70.3a	1.7 ± 70.7a

*Different lowercase letters within each column indicate significant differences between ID, CK, and EI at P < 0.05 based on the t-test.*

### Gene Expression Profiles of *A. catechu* Seedlings Under Different Iron Levels

To identify the key genes involved in the response to either ID or EI in *A. catechu* seedlings, transcriptome analysis was performed. The cDNA libraries for ID, EI and CK were constructed, respectively, and 64.27G clean data was generated (Supplementary Table 3). Totally 278,541 transcripts with the average length of 1,251 bp were obtained after assembly. Finally, 224,186 unigenes were obtained with the N50 of 2,461 bp (Supplementary Tables 4–6). The raw data of transcriptome sequencing has been deposited to the NCBI Short Reads Archive (SRA) with the accession number PRJNA695119. According to the annotation of unigenes against the Nr database, a high percentage of *A. catechu* transcripts were closely matched to the sequences of *Elaeis guineensis* (54.65%), *Phoenix dactylifera* (27.37%), and *Cephalotus follicularis* (1.5%) (Supplementary Figure 5).

**FIGURE 4:**
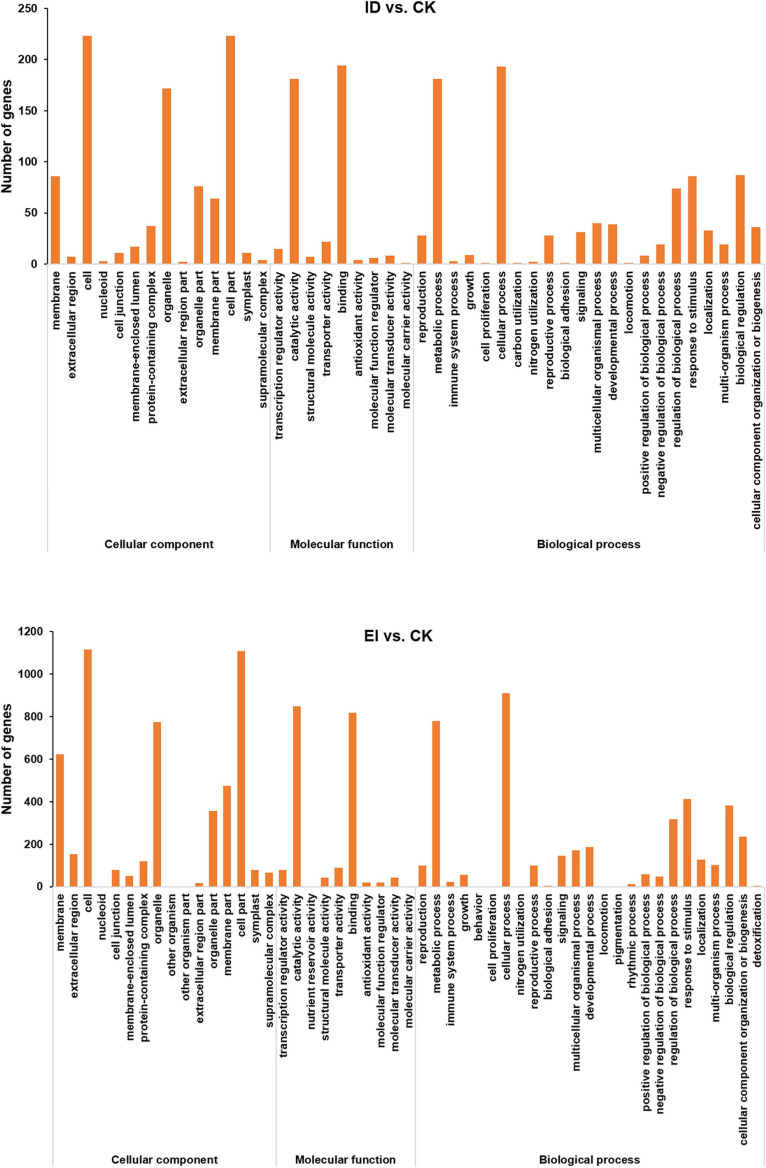
Summary of GO (gene ontology) categories of DEGs. The numerals beside the histogram indicate the number of DEGs.

Digital Gene Expression Profiling (DGE) analysis identified 454, 1,915, and 2,102 differentially expressed genes (DEGs) from ID vs. CK, EI vs. CK and ID vs. EI, respectively (Supplementary Figure 6). The functions of DEGs were classified according to the Gene Ontology (GO) classifications. In total, 454 DEGs from ID vs. CK were enriched into GO pathways, including the cell part, binding, cellular process, catalytic activity, metabolic process, and other functional categories. Between EI and CK, there were 1915 GO-annotated DEGs, which were mainly categorized into the cell part, cellular process, catalytic activity, binding, metabolic process, organelle, and other functional categories (Figure 4).

**FIGURE 5:**
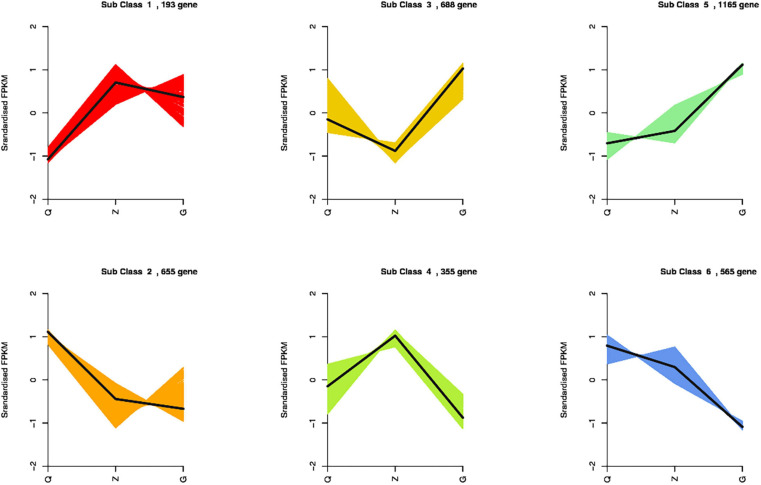
*K*-means clustering analysis of the DEGs into six clusters according to their expression profiles. The cluster names and the number of unigenes for each cluster are indicated.

Totally 3,621 DEGs were identified from all the combinations. Thesegenes were classified into six groups according to their expressionpatterns (Figure 5). We observed that the genes encoding chlorophyll *a-b* binding protein, photosystem I and II protein, ferredoxin, ferritin, metallothionein, fatty acid desaturase, celloluse synthase and flavonoid 3′,5′-hydroxylase showed consistently up-regulation along with the increasing iron level, while the genes encoding chloroplast processing peptide, cysteine-rich receptor protein kinase, wall-associated receptor kinase, early light-induced protein, ferredoxin-dependent glutamate synthase, and glutamate synthase showed consistently down-regulation along with the increasing iron level. Specifically, the genes encoding dehydrogenase, aspartic proteinase, E3 ubiquitin, proteasome activator, protein detoxification and plastid-lipid-associated protein showed up-regulation, and the genes encoding nitrate reductase and chloroplast enhancing stress tolerance protein showed down-regulation in the samples under ID conditions. Moreover, the genes encoding anthocyanidin, expansin, pectin, GDSL esterase, peroxidase, squalene monooxygenase, sucrose synthase, pentatricopeptide repeat-containing protein, protein phosphatase 2C, and cell number regulator showed up-regulation in both ID and EI samples, while the genes encoding actin-related protein, auxin response factor, nitrogen regulatory protein, lipoxygenase, and vacuolar-sorting receptor showed down-regulation in both ID and EI samples (Additional Files 1–3).

### Transcription Factors Analysis

**FIGURE 6:**
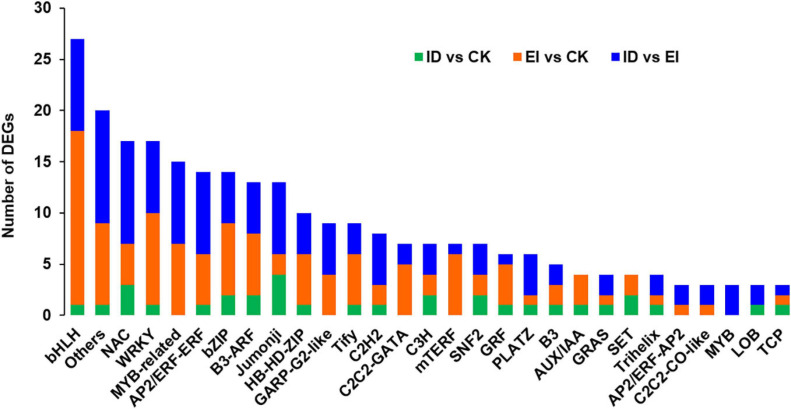
Analysis of transcription factors (TFs) and transcriptional regulators (TRs) in ID, CK and EI.

The DEGs encoding transcription factors were selected and analyzed separately (Figure 6). The results showed that 34 TF (transcription factor)-encoding genes belong to 24 TF or TR (transcription regulator) families were identified between ID and CK. Among them, 1 gene encoding bHLH93 TF showed up-regulation, 1 gene encoding WRKY TF showed down-regulation, and 3 genes encoding NAC TFs showed up-regulation (1 of 3) and down-regulation (2 of 3). Totally 120 TF-encoding genes belong to 34 TF or TR families were identified between EI and CK. The gene family with the highest number of DEGs was bHLH (14.2%, 15 were up-regulated and 2 were down-regulated), followed by WRKY (7.5%, 9 were down-regulated), bZIP (7.5%, 5 were up-regulated and 2 were down-regulated) and NAC (4 were down-regulated). Totally 129 TF-encoding genes belong to 41 TF or TR families were identified between EI and ID. The gene family with the highest number of DEGs was NAC (7.8%, 10 were up-regulated), followed by bHLH (7.0%, 7 were down-regulated and 2 were down-regulated), MYB (6.2%, 4 were up-regulated and 4 were down-regulated) and AP2/ERF (5 were up-regulated and 3 were down-regulated).

### Metabolome Analysis in *A. catechu* Seedlings Under Different Iron Levels

In order to reveal the variation of metabolites in *A. catechu* seedlings grown under different iron level, we performed metabolome analysis. Totally 106 metabolites showed significant difference between ID and CK samples, and 69 and 37 of them showed higher and lower content in ID samples than that in CK samples, respectively. Among them, the content of naringenin, butin and hesperetin was increased by more than threefold, while the content of xanthohumol, purine and N-p-coumaroylspermine was decrease by approximately 60%. Totally 116 metabolites showed significant difference between EI and CK samples, and 43 and 73 of them showed higher and lower content in EI samples than that in CK samples, respectively (Supplementary Figure 6). Among them, the content of 1,5-Anhydro-D-glucitol increased by more than 800-fold, the content of oxalic acid and nicotinic acid-hexoside increased by more than fourfold, while the content of 5-O-p-coumaroyl shikimic acid O-hexoside, 2-aminoethanesulfonic acid and 5-O-p-Coumaroyl shikimic acid showed more than 90% decrease (Additional Files 4–6).

**FIGURE 7:**
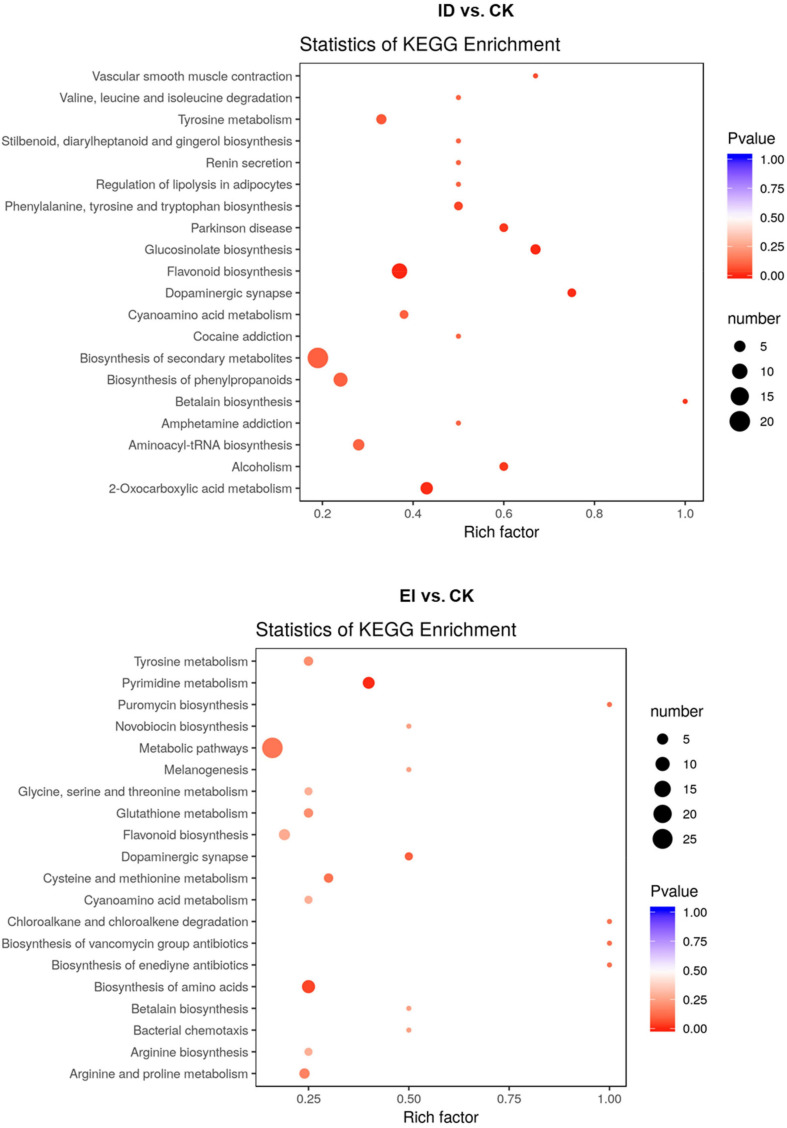
Scatter plot analysis of the DAMs in response to the ID and EI treatment in *A. catechu* seedlings leaves.

All the differentially accumulated metabolites (DAMs) were classified into different pathways through KEGG analysis. The 106 DAMs from ID vs. CK were classified into 105 pathways, such as biosynthesis of secondary metabolites (ko01100), flavonoid biosynthesis (ko00941) and biosynthesis of phenylpropanoids (ko00940). The 116 DAMs from EI vs. CK were classified into 72 pathways, such as metabolic pathways (ko01100), biosynthesis of secondary metabolites (ko01100), biosynthesis of amino acids (ko01230) and flavonoid biosynthesis (ko00941) (Figure 7).

### Overview of the Correlations in Metabolomics and Transcriptomics

**FIGURE 8:**
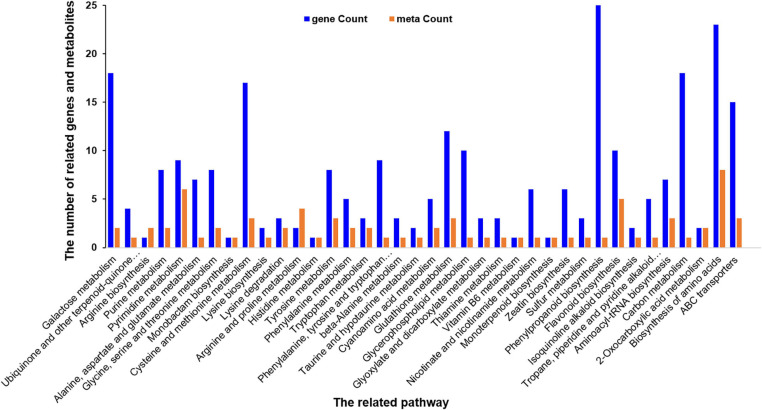
Histogram of the differentially expressed related genes and metabolites in response to ID in *A. catechu* seedlings leaves.

The correlation between metabolomics and transcriptome data was analyzed in order to further understand the response of *A. catechu* to ID. Among 454 DEGs, the expression profiles of 178 have positive correlation with the content profiles of 109 metabolites, such as p-Coumaryl alcohol, L-phenylalanine, naringenin and butin, between ID and CK. The differentially expressed genes and metabolites were both enriched in some KEGG pathways, including phenylalanine metabolism, biosynthesis of amino acids, pyrimidine metabolism, ABC transporters and glutathione metabolism (Figure 8).

### Nitrogen Assimilation Was Impacted by Iron Deficiency in *A. catechu* Seedlings

**FIGURE 9:**
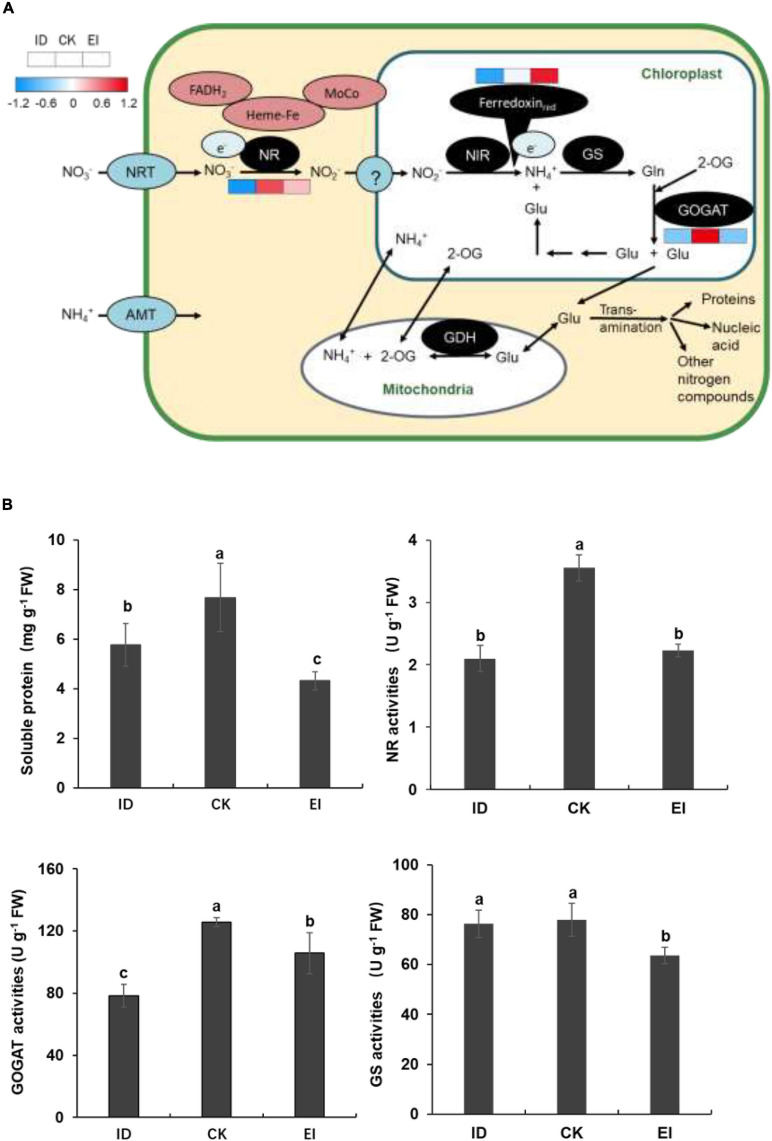
Nitrogen assimilation pathway and activities of key enzymes related with N metabolism in ID, CK, and EI leaves. **(A)** Pathway viewer of main N metabolism in *A. catechu* leaf. Heat maps were drawn using *log2*-transformed FPKM values. **(B)** Comparison of soluble protein, Nitrate reductase (NR), glutamate synthase (GOGAT), and glutamine synthetase (GS). The small bars show standard deviation. Different letters represent significant differences at *P* < 0.05 according to Duncan’s multiple range tests.

The element analysis showed that the nitrogen level was significantly reduced in the seedlings grown under ID. Moreover, the content of most free amino acids was elevated (Supplementary Figure 7), and the genes encoding key enzymes involved into the nitrogen assimilation pathway showed consistent down-regulation indicated by metabolome and transcriptome data. We speculated that ID could affect the nitrogen assimilation in *A. catechu*, therefore, the genes and metabolites involved into this pathway were further studied. We observed that although the expression level of genes encoding ferredoxin up-regulation along with the iron levels increasing, the expression of *NR* (*nitrate reductase*) and *GOGAT* (*glutamate synthase*) genes showed down-regulation in both ID and EI conditions (Figure 9A). This result implied that ID and EI could impact the iron-dependent nitrogen assimilation, therefore resulted in chloroplast degeneration. Consistently, the enzymatic activity of NR and GOGAT, as well as the soluble protein content, was reduced under both ID and EI condition (Figure 9B).

### Phenylpropanoid and Flavonoid Biosynthesis Were Provoked by ID

**FIGURE 10:**
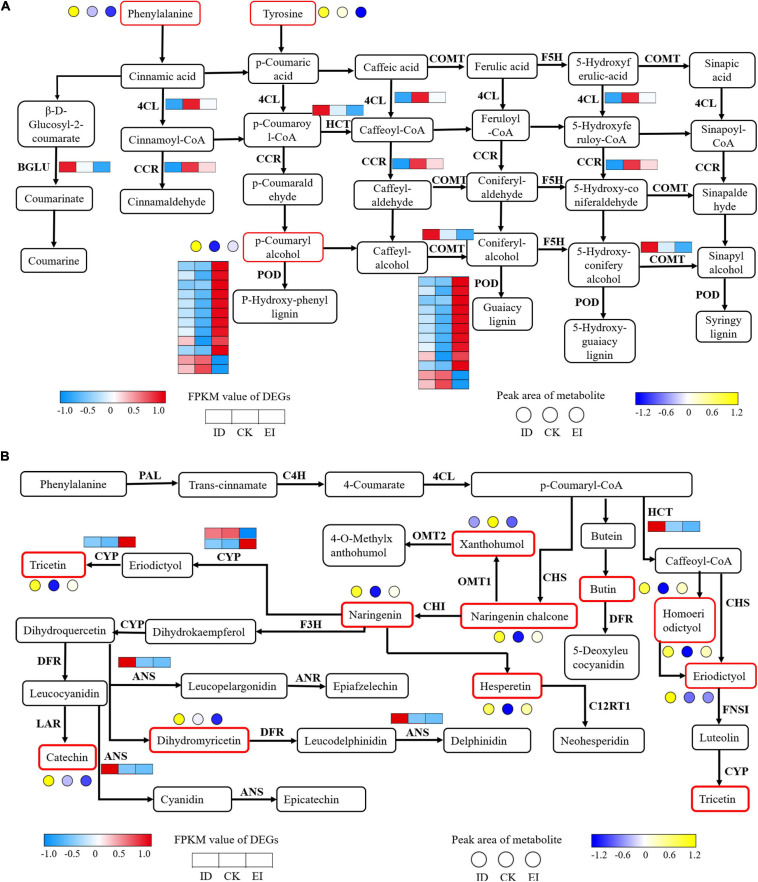
Transcriptomic and metabolomic variation related to phenylpropanoid **(A)** and flavonoid **(B)** metabolism. 4CL, 4-coumarate CoA ligase; BGLU, beta-glucosidase; CCR, cinnamoyl-CoA reductase; COMT, caffeic acid 3-O-methyltransferase; F5H, flavanone 5-hydroxylase; PAL, phenylalanine ammonia-lyase; C4H, cinnamic acid 4-hydroxylase; CHS, chalcone synthase; CHI, chalcone isomerase; F3H, flavanone 3-hydroxylase; DFR, dihydroflavonol 4-reductase; ANS, anthocyanidin synthase; FLS, flavonol synthesis; ANR, anthocyanin reductase.

The strategy of *A. catechu* seedlings to survive the ID conditions was implied by the present results. We identified 16 DEGs involved into the phenylpropanoid biosynthesis pathway. Among them, the genes encoding 4CL (4-coumarate–CoA ligase) and CCR (cinnamoyl-CoA reductase) showed significant down-regulation in both ID and EI. The genes encoding BGLU (beta-glucosidase), HCT (shikimate O-hydroxy cinnamoyl transferase), and COMT (caffeic acid 3-O-methyltransferase) showed significant down-regulation in ID but significant up-regulation in EI. In addition, most genes encoding POD (peroxidase) showed significant up-regulation in EI (Figure 10A). For the flavonoid biosynthesis pathway, 2 genes encoding ANS (anthocyanidin synthase) and HCT (shikimate O-hydroxy cinnamoyl transferase), respectively, showed significant up-regulation under ID. Totally 10 metabolites, including catechin, naringenin, xanthohumol, eriodictyol, hesperetin, dihydromyricetin, naringenin chalcone, tricetin, homoeriodictyol, and butin, also showed significant increased content in ID samples (Figure 10B).

### qPCR Analysis on Selected Key Genes

Totally 27 genes were selected according to the previous analysis forconfirming their expression patterns using qPCR. The results showed that the genes encoding 4CL, GOGAT and NRT showed down-regulation in both ID and EI. On the contrary, the genes encoding bHLH transcription factor and expansin showed significant up-regualtion in both ID and EI (Supplementary Figure 8). The gene encoding ferritin, WRKY70 and fatty acid export protein were up-regulated, while the gene encoding plant cysteine oxidase, FRO2, isoflavone 2′-hydroxylase and chloroplast processing peptidase were down-regulated along with the increasing iron level (Supplementary Figure 8). The qPCR result has a significant correlation with the RNA-seq data (Supplementary Figure 9).

## Discussion

There are many different causes of leaf chlorosis in plants. While in this study, iron deficiency was proved to be the main determinant for leaf chlorosis in *A. catechu*. The iron deficiency in the soil of *A. catechu* planting area might due to the low availability of the transition from ferric to ferrous (Honetschlägerová et al., 2018). Iron has been extensively documented for its role in chlorophyll biosynthesis (Hänsch and Mendel, 2009). Iron level could also regulate the formation rate of aminoacetylalanine (ALA), which is the precursor for not only chlorophyll but also heme (Pushnik and Miller, 1989). Iron is necessary for the formation of protochlorophyll from magnesium protoporphyrin (Marschner and Marschner, 2012). Moreover, there are 20 iron atoms directly involved in the electron transport chain in the thylakoid membrane. The photosystem I is a strong iron sink (12 iron atoms per complex) compared with PS II (3 iron atoms per complex) and Cyt *bf* complex (5 iron atoms per complex) (Raven et al., 1999). The high requirement of iron for the structural and functional integrity of thylakoid membrane, and the extra iron for ferredoxin and the biosynthesis of chlorophyll could explain the particular sensitivity of chloroplasts, especially endosomes, to iron deficiency. We observed that iron deficiency resulted in chloroplast degeneration in a short period, indicating that iron deficiency also impacted the development of chloroplast. We noticed that the *A. catechu* leaves were very sensitive to iron deficiency, while have relatively strong tolerance to the deficiency of other elements (nitrogen, zinc, magnesium, boron) (Supplementary Figures 1–3). These evidences confirmed that iron is one of the critical determinants for physiological chlorosis.

Since iron deficiency is verified to be the main reason for leaf chlorosis, iron supplement was supposed to be an efficient way to recover the *A. catechu* seedlings. We observed obvious dark green leaves in the seedlings grown under excessive iron conditions. It may be related to the promotion of chlorophyll synthesis by EI at the early stage of treatment. However, threefold of normal iron level also resulted in abnormal chloroplast structure, reduced net photosynthetic rate and decreased biomass. This result could be attributed to the high iron toxicity. The toxic damage of iron is caused by its reaction with hydrogen peroxide (H_2_O_2_) and form hydroxyl radical (OH^–^), which is the most active reactive oxygen species (ROS) (Dixon and Stockwell, 2014). The ROS can directly damage cells by destroying biological molecules such as lipids or proteins (Li B. et al., 2019). During the long-term evolution of plants, antioxidant enzymes that scavenge free radicals have been produced. Studies have shown that the activity of POD (peroxidase) enhances with the increase of reducing iron concentration (Wu et al., 2014). On the one hand, POD scavenges H_2_O_2_ in plants, and prevents Fe^2+^ from entering cells by accelerating cell wall lignification (Wu et al., 2014). In our transcriptome data, most genes encoding POD (10 of 12) were significantly up-regulated under the high iron condition, indicating that the *A. catechu* seedlings were improving the cellular defense mechanism to fight against iron toxicity. In rice (*Oryza sativa*), iron toxicity initiated when the iron concentration was more than 300 mg/kg, and serious iron toxicity occurred when the iron concentration is more than 500 mg/kg (Yamauchi, 1989), which is much higher than the concentration adopted in this study. In addition, although the iron content in the subterranean parts was largely increased in the seedlings grown under EI, the iron content in aerial parts was even reduced, implying that there is a defend mechanism to prevent excessive iron transport to *A. catechu* leaves. These results proved that the range of iron level suitable for the growth of young *A. catechu* seedlings is very narrow, and confirmed again that *A. catechu* is an iron-sensitive plant species.

An intriguing hypothesis proposed that some proteins might be used as a source of amino acids, carbon skeletons and N-NH_4_^+^ under iron deficiency through an iron-regulated protein degradation machinery (Donnini et al., 2010). In our study, soluble protein content was significantly decreased, and the free amino acid content was largely increased indicated by the metabolome data. The increased amino acids content was in agreement with the down-regulation of *NR* genes, which are iron-dependent (Campbell, 1999; Borlotti et al., 2012). NR is a cytoplasmic enzyme consist of two identical subunits. Each subunit contains three cofactors covalently binding to NR, including adenine dinucleotide (FAD), Heme (heme-Fe), and molybdopterin (a molybdenum containing co-factor). Therefore, the reduction of NR activity might be attributed to both transcription suppression and heme deactivation.

Besides NR, genes encoding GOGAT were also consistently down-regulated by ID. GOGAT is involved in ammonium assimilation. It catalyzes the transfer of amide group (−NH_2_) from glutamine to 2-oxoglutarate. The conversion of glutamine to glutamate takes place in plastids which have two isoforms of GOGAT. The ferredoxin-linked GOGAT isoform dominates in leaves, which contains an Fe-S cluster transferring electrons during the reductive synthesis of two glutamate molecules from one 2-oxoglutarate and one glutamine molecule. Similar to the case in NR, ID reduced both the transcription level of *GOGAT* and the activity of Fe-S protein. Therefore, our data demonstrated that ID inhibited nitrogen assimilation in *A. catechu* leaves, similar results of which has recently been reported in fragrant citrus (Jin et al., 2017) and soybean (Chu et al., 2019).

The accumulation of flavonoids and flavonols, and the up-regulation of genes involved into the flavonoids and flavonols biosynthesis pathway was assumed to be a critical strategy for *A. catechu* seedlings to survive ID. Plants grown under ID often secrete small molecule organic compounds, including organic acids, phenols and flavonoids, into the rhizosphere to improve the availability of iron (Rodriguez-Celma et al., 2011). For example, dicotyledons such as *Beta vulgaris* and *Medicago truncatula* can secrete flavonoids to reduce trivalent iron in soil to divalent iron with high solubility to improve the availability of iron (Brumbarova et al., 2014). The root exudates, such as low molecular weight organic acids including citric acid, malic acid and oxalic acid, could activate insoluble iron through chelation or acidify rhizosphere to promote iron absorption (Jones and Darrah, 1994). Another kind of compounds secreted by root under ID is phenols, which can directionally reconstruct the microbial community structure of rhizosphere soil, thus indirectly improve the availability of iron. Jin et al. (2006) found that phenolic compounds secreted under ID in red clover can inhibit the growth and reproduction of phenol sensitive microorganisms in rhizosphere soil through its antibacterial function. The phenolic compounds can also be used as carbon sources to promote the growth and reproduction of phenol resistant microorganisms. The combined action of these two aspects can form a dominant microbial community which can secrete siderophore in the rhizosphere (Jin et al., 2014). Our data demonstrated that most genes involved in the process of decarboxylation of phenolic acids were also down-regulated in the seedlings grown under ID. The inhibition of decarboxylation activity could result in the accumulation of phenolic acids, which might be an important compound for *A. catechu* seedlings to deal with ID. On the other hand, the phenylalanine pathway is identified as the major target for *A. catechu* to response to ID. The genes including *HCT*, *ANS*, *BGLU*, and *COMT*, and the metabolites including catechin, naringenin, xanthohumol, eriodictyol, hesperetin, dihydromyricetin, naringenin chalcone, tricetin, homoeriodictyol, and butin were important candidates for indicating the ID status of *A. catechu* seedlings.

The bHLH family transcription factors were identified from both ID vs. CK and EI vs. CK. It is known that at least 16 bHLH transcription factors are closely involved into the regulation of plant iron homeostasis through a complex regulatory network (Gao et al., 2019). It was found in Arabidopsis that the *FIT* (*FER-LIKE FE DEFICIENCY-INDUCED TRANSCRIPTION FACTOR*) gene could form a heterodimer with bHLH038, bHLH039, bHLH100, and bHLH101, respectively, and activate the expression of downstream target genes such as *FRO2* and *IRT1* under iron deficiency (Sivitz et al., 2012). The expression of bHLH11 was significantly inhibited under iron deficiency in Arabidopsis (Tanabe et al., 2018). A number of genes encoding WRKY were identified from ID vs. CK. The WRKY transcription factors have been reported to play a role in response to nutrient deficiency (Kasajima et al., 2010; Ding et al., 2013; Su et al., 2015). The WRKY-related cis-element, W-box, was located in the promoter sequences of *IRT* and *NAS4*, which are critical genes response to iron deficiency. A *WRKY46* gene from Arabidopsis was verified to promote Fe translocation from root to shoot by suppressing the expression of a nodulin-like gene (*VIT1-like1*) under iron deficiency (Yan et al., 2016). Moreover, a gene encoding an IDEF2 transcription factor from rice was proved to response to ID. IDEF2 can recognize the iron deficiency-responsive *cis*-acting element 2 (IDE2) thus regulate the genes involved in iron homeostasis. Knocking down of *IDEF2* resulted in abnormal Fe allocation between the shoots and roots (Li Q. et al., 2019). These evidences indicated that the DEGs encoding bHLH, WRKY and NAC identified from our transcriptome data, might be key regulators in the chlorosis process of *A. catechu*.

## Conclusion

In summary, iron was identified as a determinant for leaf chlorosis in *A. catechu* seedlings. The *A. catechu* seedlings have good adaptability to different nutrients (nitrogen, magnesium, zinc, boron) except for iron. Iron deficiency seriously impacted chloroplast development and nitrogen assimilation in *A. catechu* leaves. *A. catechu* seedlings response to ID through alter the flavonoids and flavonols biosynthesis, and secrete flavones and phenolic acids to the rhizosphere. The transcription factors bHLH, WRKY, and NAC were identified as key regulators under ID. On the other hand, high iron is also deleterious to *A. catechu* seedlings. An unknown mechanism blocks the iron transport and retains iron in the subterranean parts of the plants. The up-regulation of POD-related genes was assumed to be a defense strategy against the excessive iron toxicity. These evidences indicated that *A. catechu* is an iron-sensitive species, therefore we suggest that the precise iron control, including the detection of iron level, the way of iron supplement and the maintenance of suitable iron level, should be taken into account for *A. catechu* cultivation in the future.

## Data Availability Statement

The datasets presented in this study can be found in online repositories. The names of the repository/repositories and accession number(s) can be found in the article/Supplementary Material.

## Author Contributions

JL: conceptualization, methodology, software, investigation, and writing—original draft. XC: investigation and data curation. XJ: software and writing—review. LL: resources and funding acquisition. HC: software. WQ: resources and formal analysis. ML: data curation and writing—review and editing. All authors contributed to the article and approved the submitted version.

## Conflict of Interest

The authors declare that the research was conducted in the absence of any commercial or financial relationships that could be construed as a potential conflict of interest.

## Publisher’s Note

All claims expressed in this article are solely those of the authors and do not necessarily represent those of their affiliated organizations, or those of the publisher, the editors and the reviewers. Any product that may be evaluated in this article, or claim that may be made by its manufacturer, is not guaranteed or endorsed by the publisher.
