# Supplementary material for: Iron Deficiency Leads to Chlorosis Through Impacting Chlorophyll Synthesis and Nitrogen Metabolism in Areca catechu L
Source: Front Plant Sci. 2021 Aug 2;12:710093. doi: 10.3389/fpls.2021.710093 (PMC8365612; doi:10.3389/fpls.2021.710093)
Supplement: Supplementary file 7 [file Data_Sheet_7.docx]

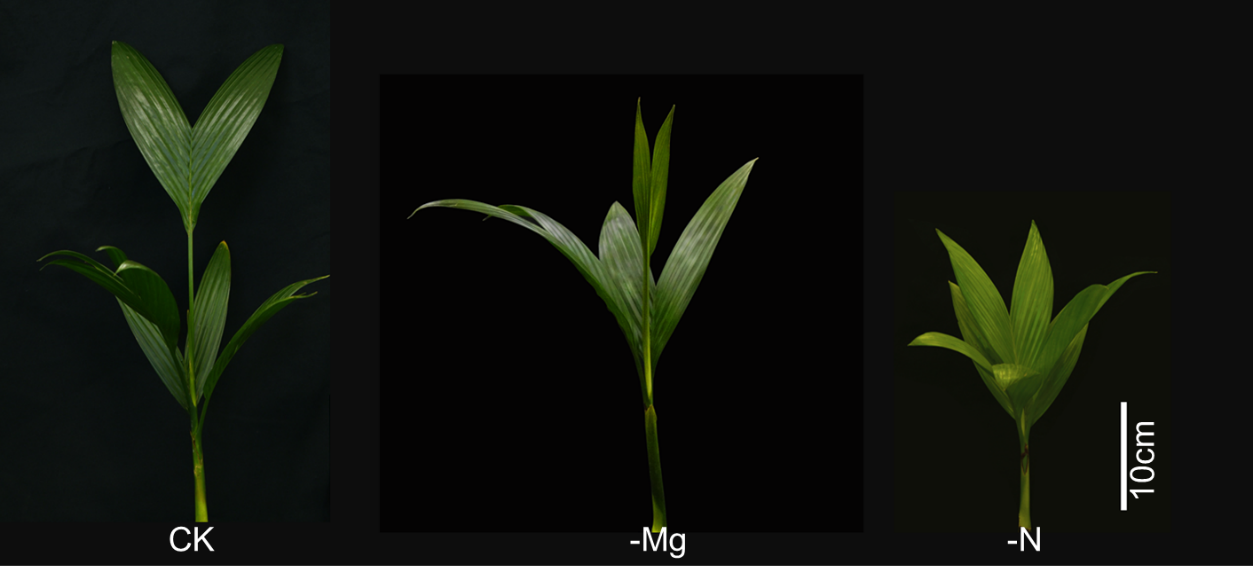


**Fig S1.** Morphological characteristics of *A. catechu* seedlings at 80 days after N and Mg deficient and normal treatments. -N (nitrogen deficient, 0 N), -Mg (magnesium deficient, 0 Mg), CK (normal treatment).


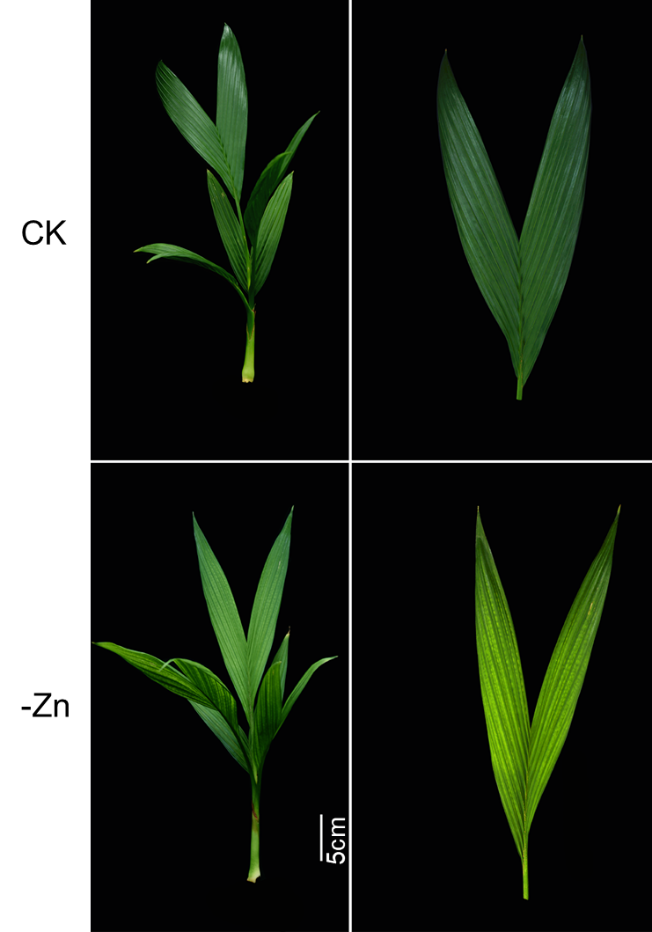


**Fig. S2.** Morphological characteristics of *A. catechu* seedlings at 80 days after Zn deficient（0 Zn）and normal treatments (CK).


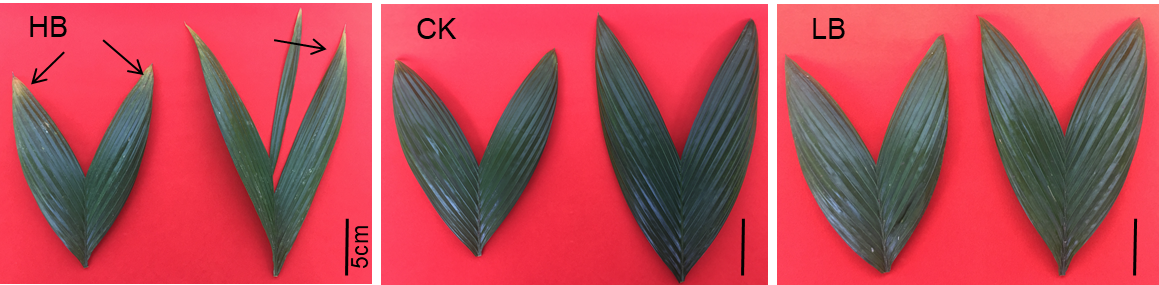


**Fig. S3.** Morphological characteristics of *A. catechu* seedlings under different B concentrations. The seedlings were treated more than 80 d. LB (Low boron concentration, 0 H_3_BO_3_), CK (normal boron concentration, 50 μmol/L H_3_BO_3_), HB (High boron concentration, 150 μmol/L H_3_BO_3_).


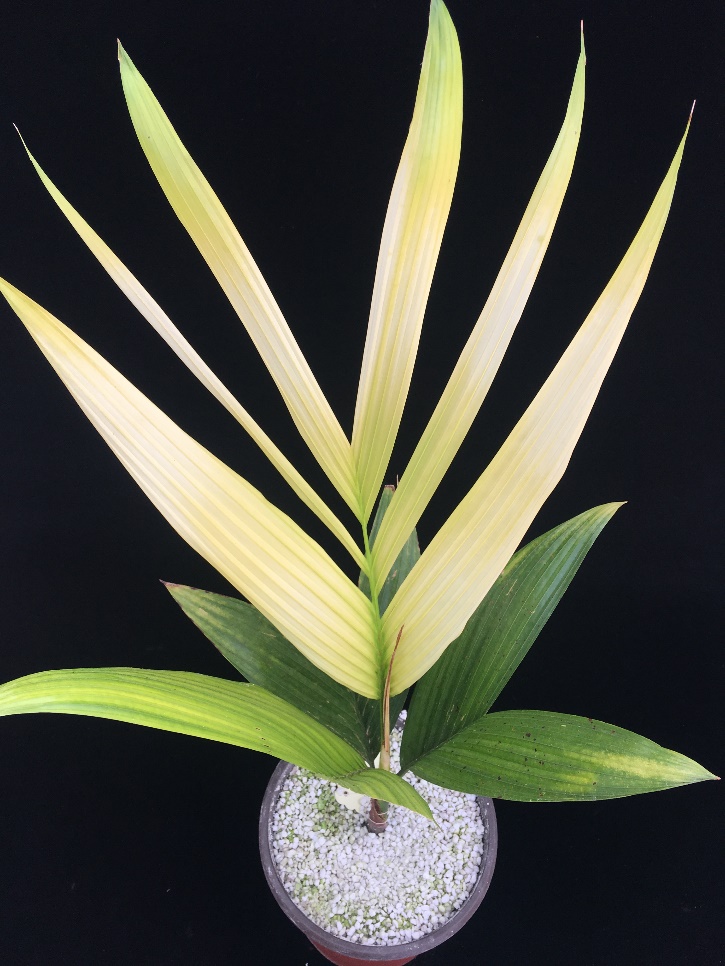


**Fig. S4** Morphological characteristics of *A. catechu* seedlings at 40 days after ID treatment.


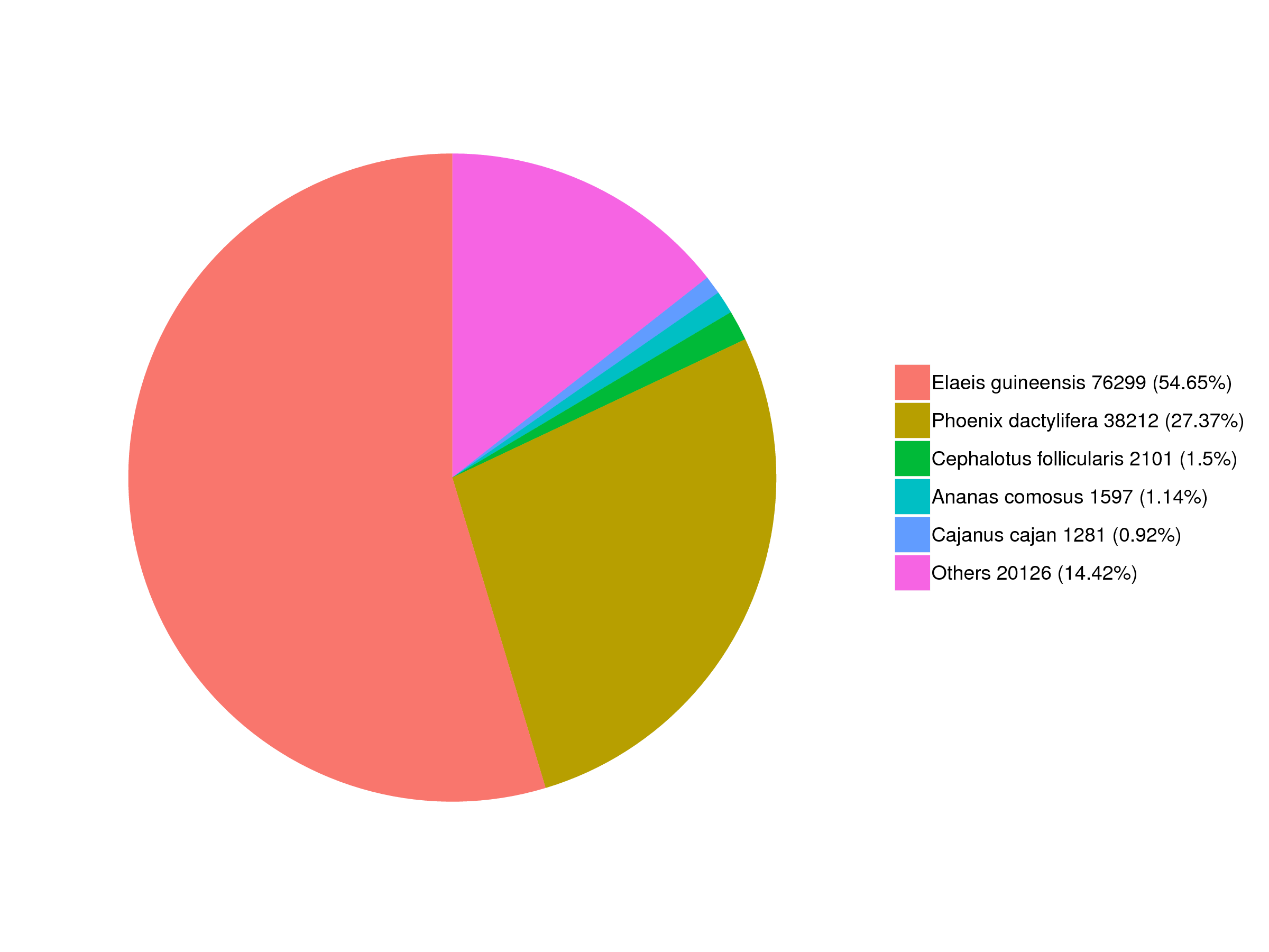


**Fig. S5.** Species classification of annotated unigenes of arecanut transcriptome

**a b**

**
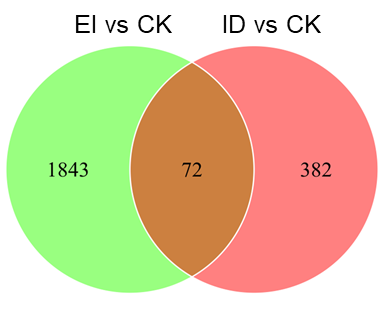

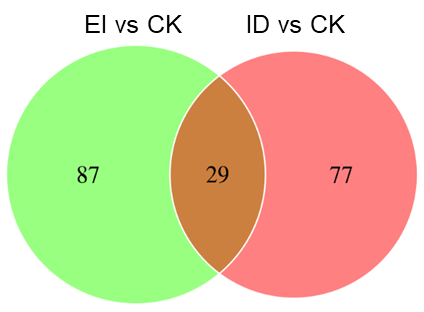
**

**Fig. S6.** The differentially expressed genes (DEGs) and metabolites (DEMs) in ID, CK and EI. a, Venn diagram of DEGs. b, Venn diagram of DEMs


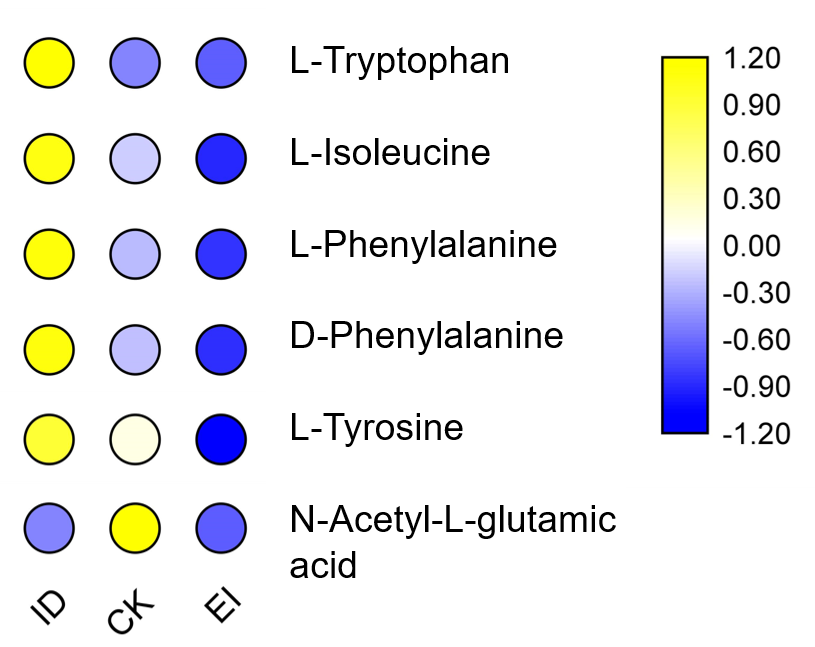


**Fig. S7.** Variation of free amino acids in ID, CK and EI.

**
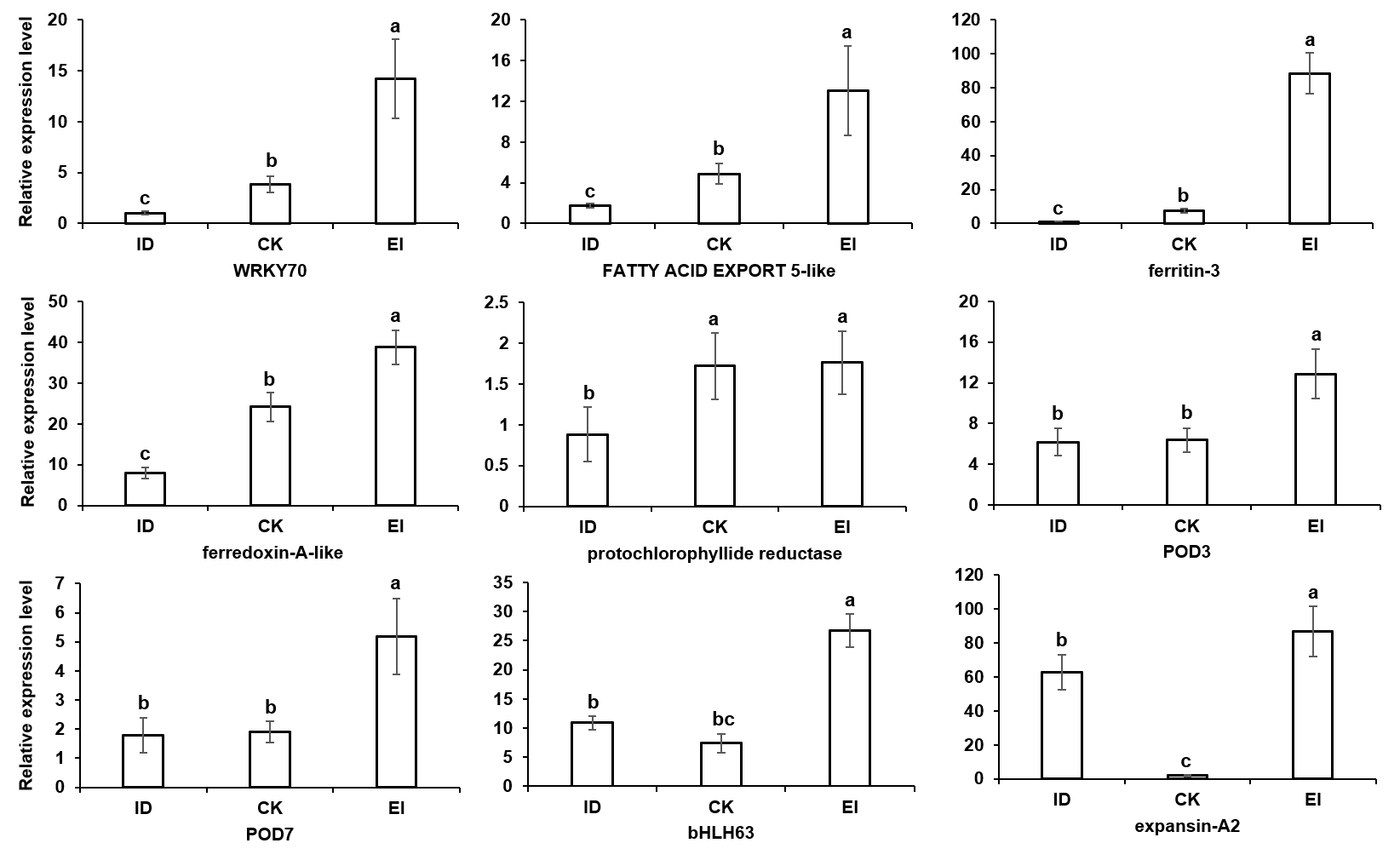
**

**
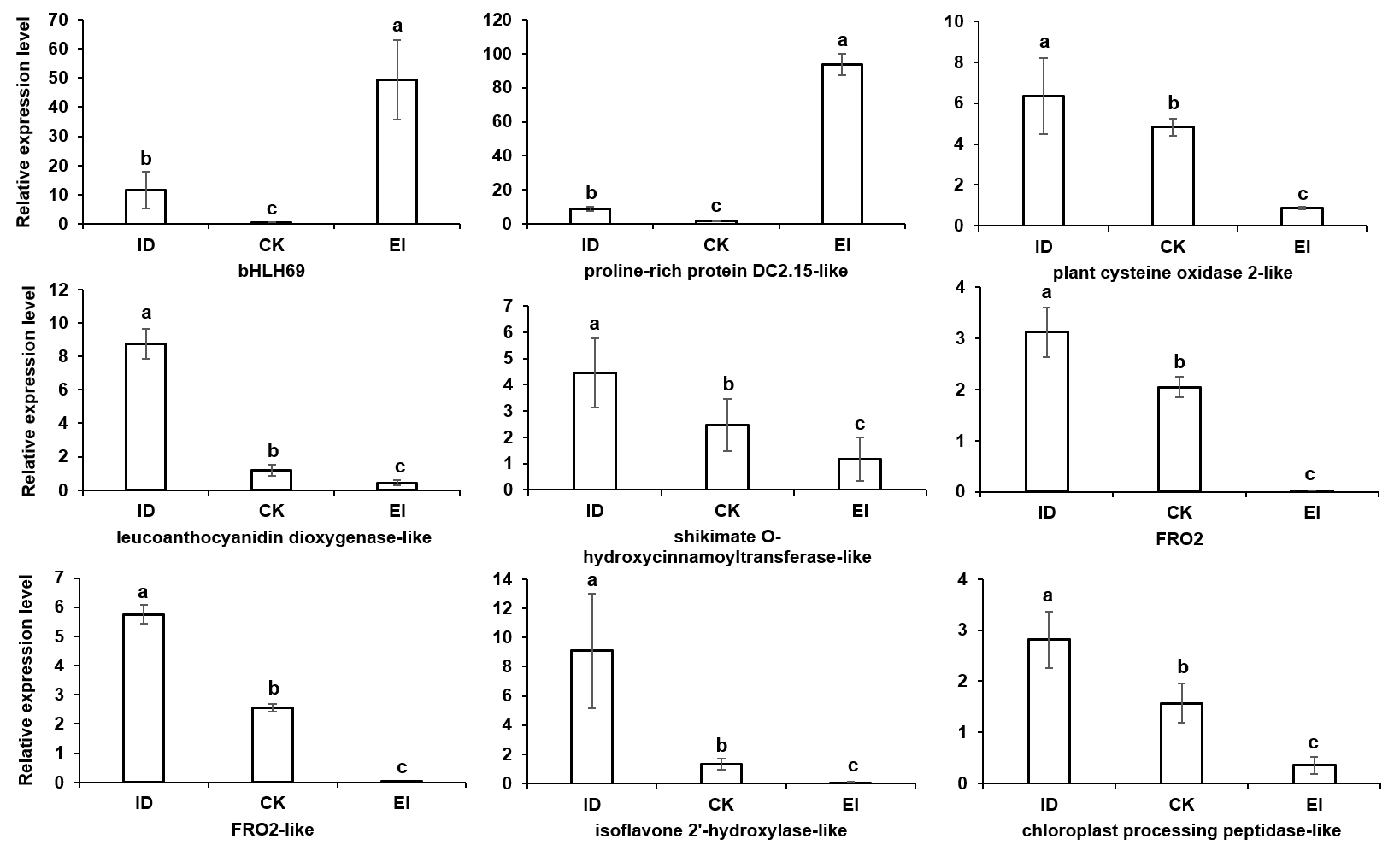
**

**
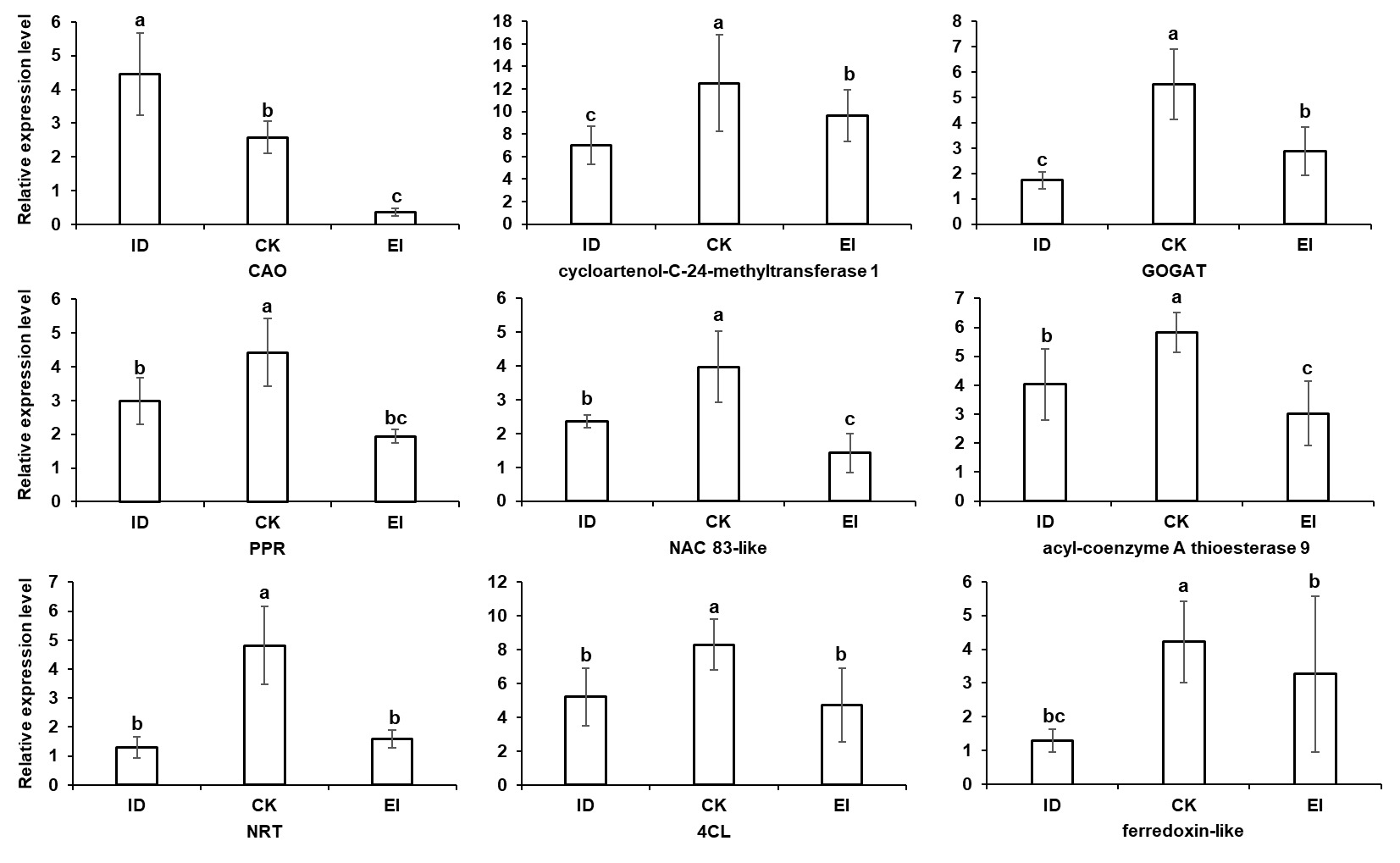
**

**Fig. S8.** Quantitative real-time PCR (RT-qPCR) analysis of the expression of twenty-seven DEGs in ID, CK and EI leaves.

**
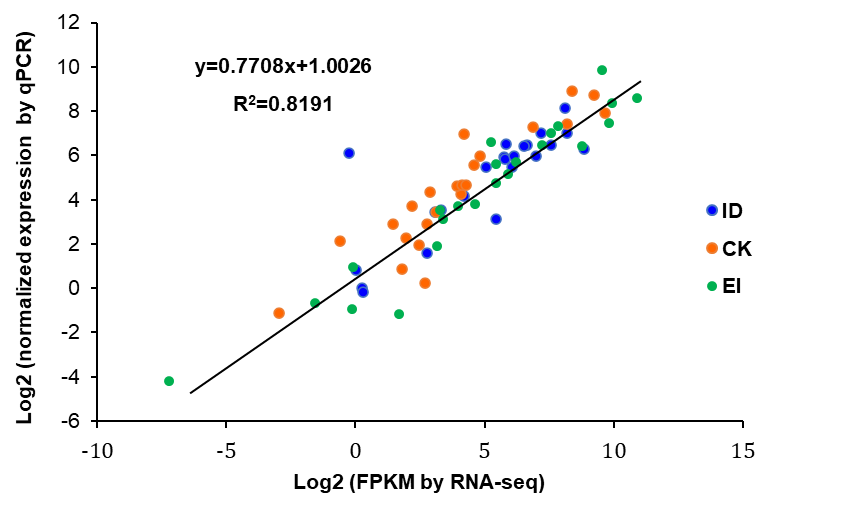
**

**Fig. S9.** Correlation of gene expression results. The x-axis represents the value of Log2 FPKM and the y-axis represents the value of Log2 normalized expression level. Blue round dot represent ID leaves. Orange round dot represent CK leaves. Green round dot represent EI leaves. R^2^ value represent the correlation between RNA-seq and qPCR results.
