# Supplementary material for: Iron Deficiency Leads to Chlorosis Through Impacting Chlorophyll Synthesis and Nitrogen Metabolism in Areca catechu L
Source: Front Plant Sci. 2021 Aug 2;12:710093. doi: 10.3389/fpls.2021.710093 (PMC8365612; doi:10.3389/fpls.2021.710093)
Supplement: Supplementary file 8 [file Data_Sheet_8.docx]

**Table S1.** Information of the target genes and relevant primers using in this study

| **Gene** | **Functional annotation** | **Primer (5’ to 3’)** |
| --- | --- | --- |
| Cluster-20636.63062-F | CAO | cagccatatcaccccaacctgaa |
| Cluster-20636.63062-R |  | gcacacaacctggcttccca |
| Cluster-20636.77655-F | chloroplast processing peptidase-like | cgtgacgaatctgactgttgggtt |
| Cluster-20636.77655-R |  | tgtttgctgcgagaacggga |
| Cluster-20636.97835-F | NRT | tggcgcaagaccgacgacaacctc |
| Cluster-20636.97835-R |  | caaccacgtatgcccgaacgaagt |
| Cluster-20636.129709-F | isoflavone 2'-hydroxylase-like | tgttccgcgaatccggtgga |
| Cluster-20636.129709-R |  | actcctcggagcttgccatg |
| Cluster-20636.93128-F | NAC83-like | tcgacgggctgcggttactg |
| Cluster-20636.93128-R |  | cgaggcgatactcgtgcatgacc |
| Cluster-20636.92228-F | PPR | gatcgccaactacgcaaaccgt |
| Cluster-20636.92228-R |  | gtcgctgacttgtgggaatgc |
| Cluster-20636.98274-F | GOGAT | agcaccttgcaaatcgacctgtga |
| Cluster-20636.98274-R |  | tagccccaagagacatgccacc |
| Cluster-20636.118277-F | shikimate O-hydroxycinnamoyltransferase-like | gatgccgatctacgaggccgactt |
| Cluster-20636.118277-R |  | cgcctcaaatgacacaatcaccga |
| Cluster-20636.78456-F | leucoanthocyanidin dioxygenase-like | atgatcgccgggtacgggagcaa |
| Cluster-20636.78456-R |  | cacctcaacgtagtcggcaggtt |
| Cluster-20636.116983-F | 4CL | atccggaggcaacggaacggac |
| Cluster-20636.116983-R |  | tgagcatggcttcgagttcagc |
| Cluster-20636.19713-F | FRO2 | ggttgtgaggtggcctctcggaa |
| Cluster-20636.19713-R |  | tggattgccacaatttctcgcct |
| Cluster-20636.100830-F | acyl-coenzyme A thioesterase 9 | ttgttggcagaggggcgtatctt |
| Cluster-20636.100830-R |  | gctaggttcatcaaaaagccccca |
| Cluster-20636.64604-F | cycloartenol-C-24-methyltransferase 1 | Ctttgctcccagatggaaagggga |
| Cluster-20636.64604-R |  | ttcccgaaggggtccgccaa |
| Cluster-20636.90084-F-F | ferredoxin-like | tgtttgctgcgagaacggga |
| Cluster-20636.90084-F-R |  | agagatggatttcgacctcccgtt |
| Cluster-20636.126454-F | FRO2-like | ggttgtgaggtggcctctcggaa |
| Cluster-20636.126454-R |  | tggattgccacaatttctcgcct |
| Cluster-20636.127725-F | isoflavone 2'-hydroxylase-like | tgttccgcgaatccggtgga |
| Cluster-20636.127725-R |  | actcctcggagcttgccatg |
| Cluster-20636.525 -F | WRKY70 | tgagctcacgatgcaactccga |
| Cluster-20636.525 -R |  | gagccaccattttgaagcccgga |
| Cluster-20636.172023 -F | FATTY ACID EXPORT 5-like | tgccctgatcctcgagacggt |
| Cluster-20636.172023 -R |  | cttggaagggatatgattgccacc |
| Cluster-20636.90950-F | protochlorophyllide reductase | ttcggggcttcactttcggacc |
| Cluster-20636.90950-R |  | tggcttgctcgattgccgggga |
| Cluster-20636.64420-F | ferritin-3 | acttcgatcgggataatgtcgcac |
| Cluster-20636.64420-R |  | gacaatggatccaagcttcactc |
| Cluster-20636.49069 -F | bHLH69 | attggtggtggcactgttgcatg |
| Cluster-20636.49069 -R |  | tccccattcggcaggagacg |
| Cluster-20636.127919-F | expansin-A2 | accgctctcttcaacaacgggct |
| Cluster-20636.127919-R |  | tgtcgttggggagggcgtagttc |
| Cluster-20636.61693-F | proline-rich protein DC2.15-like | aacctcggcagtccaccgaa |
| Cluster-20636.61693-R |  | tccgcagtagttgatgagcaagct |
| Cluster-20636.57742-F | bHLH63 | aggtgtgacaacactgcagaggat |
| Cluster-20636.57742-R |  | tgcgttctccttggaggcgt |
| Cluster-20636.135022-F | POD3 | tgttaggggttgtgatgcttcggt |
| Cluster-20636.135022-R |  | agacggtatctgggcaggctttct |
| Cluster-20636.127042-F | POD7 | agcatcggcttagcacgttgct |
| Cluster-20636.127042-R |  | cctgttcggattctggacgttgag |
| Cluster-20636.30781-F | plant cysteine oxidase 2-like | tcccactgcataatcacccaggaa |
| Cluster-20636.30781-R |  | aaccttcgctagccacgcaccat |
| AcActin-F |  | attcaggtgcccggaggtcctctt |
| AcActin-R |  | gggaacatggttgatcccccacta |

**Table S2.** Element analysis of normal (CK), chlorosis (E) and slight chlorosis (SE) leaf samples collected from *A. catechu* plants grown in different areas.

|  | N  mg/g | P  mg/g | K  mg/g | Ca  mg/g | Mg  mg/g | Mn  µg/g | Fe  µg/g | Cu  µg/g | Zn  µg/g | Mo  µg/g |
| --- | --- | --- | --- | --- | --- | --- | --- | --- | --- | --- |
| CK1 | 20.36 | 1.49 | 6.12 | 5.03 | 2.42 | 64.10 | 65.59 | 4.52 | 15.46 | 0.48 |
| CK2 | 19.47 | 1.53 | 5.54 | 4.57 | 2.23 | 109.38 | 59.65 | 4.95 | 15.32 | 0.41 |
| CK3 | 17.96 | 0.99 | 4.62 | 3.59 | 1.74 | 65.75 | 68.79 | 3.60 | 17.73 | 0.40 |
| CK4 | 20.11 | 1.33 | 6.46 | 4.07 | 2.16 | 106.48 | 66.99 | 4.17 | 13.48 | 0.58 |
| CK5 | 18.10 | 1.14 | 5.29 | 2.56 | 1.80 | 85.04 | 93.99 | 4.37 | 11.01 | 0.47 |
| CK6 | 26.33 | 2.05 | 6.71 | 3.03 | 1.68 | 233.12 | 123.51 | 3.55 | 15.30 | 1.12 |
| CK7 | 21.42 | 1.89 | 5.70 | 5.03 | 1.81 | 86.69 | 83.28 | 3.01 | 22.61 | 0.90 |
| E1 | 19.51 | 1.28 | 6.10 | 3.40 | 1.53 | 63.68 | 38.79 | 3.72 | 13.16 | 0.10 |
| E2 | 20.12 | 1.51 | 7.64 | 4.28 | 2.32 | 80.16 | 44.72 | 4.63 | 17.30 | 0.62 |
| E3 | 17.59 | 1.45 | 7.74 | 3.99 | 2.07 | 123.14 | 56.22 | 4.25 | 17.61 | 0.71 |
| E4 | 15.37 | 1.74 | 8.77 | 2.41 | 1.70 | 217.35 | 46.93 | 1.73 | 8.51 | 0.36 |
| SE1 | 19.85 | 2.01 | 8.55 | 2.62 | 1.47 | 93.26 | 44.40 | 2.11 | 9.82 | 0.20 |
| SE2 | 17.33 | 1.13 | 6.26 | 3.40 | 2.04 | 56.15 | 35.22 | 3.17 | 13.54 | 0.28 |
| SE3 | 24.40 | 1.76 | 8.36 | 5.71 | 2.80 | 78.99 | 51.54 | 6.22 | 19.86 | 0.61 |
| SE4 | 23.12 | 1.87 | 7.50 | 5.01 | 1.89 | 97.19 | 56.24 | 4.57 | 15.47 | 0.40 |

**Table S3.** Summary raw data generated from *A.catechu* samples

| **Sample name** | **Raw reads** | **Clean reads** | **Clean bases** | **Error rate (%)** | **Q30 (%)** | **GC content (%)** |
| --- | --- | --- | --- | --- | --- | --- |
| ID1 | 49015464 | 44830506 | 6.72 | 0.02 | 96.03 | 48.91 |
| ID2 | 50303392 | 46721384 | 7.01 | 0.02 | 95.58 | 49.21 |
| ID3 | 48807784 | 45783984 | 6.87 | 0.02 | 96.01 | 49.6 |
| CK1 | 39816214 | 36467918 | 5.47 | 0.02 | 95.66 | 47.69 |
| CK2 | 52306522 | 48298962 | 7.24 | 0.02 | 95.84 | 48.11 |
| CK3 | 59661678 | 54791612 | 8.22 | 0.02 | 95.91 | 48.52 |
| EI1 | 42999472 | 39969910 | 6 | 0.02 | 95.94 | 48.65 |
| EI2 | 61861672 | 56638412 | 8.5 | 0.02 | 95.84 | 47.71 |
| EI3 | 58386416 | 54947688 | 8.24 | 0.02 | 95.9 | 48.56 |

**Table S4.** Distribution of transcript length interval of *A.catechu* transcriptome

| **Transcript length interval** | **200-500bp** | **500-1kbp** | **1k-2kbp** | **>2kbp** | **Total** |
| --- | --- | --- | --- | --- | --- |
| Number of transcripts | 116068 | 54919 | 51368 | 56189 | 278541 |
| Number of Genes | 62477 | 54162 | 51361 | 56186 | 224186 |

**Table S5.** Distribution of transcript length of *A.catechu* transcriptome

|  | **Mean Length** | **N50** | **N90** | **Total Nucleotides** |
| --- | --- | --- | --- | --- |
| Transcripts | 1251 | 2346 | 484 | 348382117 |
| Genes | 1488 | 2461 | 635 | 333567158 |

|  | **Number of Genes** | **Percentage (%)** |
| --- | --- | --- |
| Annotated in NR | 139616 | 62.28 |
| Annotated in KO | 109680 | 48.92 |
| Annotated in SwissProt | 90176 | 40.22 |
| Annotated in PFAM | 98323 | 43.86 |
| Annotated in GO | 98685 | 44.02 |
| Annotated in KOG | 84597 | 37.74 |
| Annotated in at least one Database | 141058 | 62.92 |
| Total Unigenes | 224186 | 100 |

**Table S6.** Gene annotation of *A.catechu* transcriptome
